# Non-coding RNA LINC00857 is predictive of poor patient survival and promotes tumor progression via cell cycle regulation in lung cancer

**DOI:** 10.18632/oncotarget.7203

**Published:** 2016-02-05

**Authors:** Lihui Wang, Yanli He, Weijun Liu, Shengbin Bai, Lei Xiao, Jie Zhang, Saravana M. Dhanasekaran, Zhuwen Wang, Shanker Kalyana-Sundaram, O. Alejandro Balbin, Sudhanshu Shukla, Yi Lu, Jules Lin, Rishindra M. Reddy, Philip W. Carrott, William R. Lynch, Andrew C. Chang, Arul M. Chinnaiyan, David G. Beer, Jian Zhang, Guoan Chen

**Affiliations:** ^1^ Guangxi Medical University, Nanning, China; ^2^ Section of Thoracic Surgery, Department of Surgery, University of Michigan, Ann Arbor, Michigan, United States of America; ^3^ Guangzhou University of Chinese Medicine, Guangzhou, China; ^4^ The First People's Hospital of Yunnan Province, Kunming, China; ^5^ Xinjiang Medical University, Xinjiang, China; ^6^ Xi'an Jiaotong University, Xi'an, China; ^7^ Department of Pathology, University of Michigan, Ann Arbor, Michigan, United States of America

**Keywords:** non-coding RNA, LINC00857, lung adenocarcinoma, prognosis, diagnosis

## Abstract

We employed next generation RNA sequencing analysis to reveal dysregulated long non-coding RNAs (lncRNAs) in lung cancer utilizing 461 lung adenocarcinomas (LUAD) and 156 normal lung tissues from 3 separate institutions. We identified 281 lncRNAs with significant differential-expression between LUAD and normal lung tissue. *LINC00857*, a top deregulated lncRNAs, was overexpressed in tumors and significantly associated with poor survival in LUAD. knockdown of *LINC00857* with siRNAs decreased tumor cell proliferation, colony formation, migration and invasion *in vitro*, as well as tumor growth *in vivo*. Overexpression of *LINC00857* increased cancer cell proliferation, colony formation and invasion. Mechanistic analyses indicated that *LINC00857* mediates tumor progression via cell cycle regulation. Our study highlights the diagnostic/prognostic potential of *LINC00857* in LUAD besides delineating the functional and mechanistic aspects of its aberrant disease specific expression and potentially using as a new therapeutic target.

## INTRODUCTION

Lung cancer is a molecularly-heterogeneous disease and the leading cause of cancer mortality [1]. The molecular basis for this clinical heterogeneity remains incompletely understood. Over the past decade, research has primarily focused on the deregulation of protein-coding genes to identify oncogenes and tumor suppressors that could serve as diagnostic and therapeutic targets for lung cancer such as *EGFR* mutation and *EML4-ALK* gene fusion [2]. Identification of oncogenic driver mutations have helped improve the outcomes in specific subtypes of patients with non-small cell lung cancer (NSCLC), however the majority of the patients with lung cancer do not have an actionable molecular aberration [3, 4]. Hence, there is an urgent need for reliable biomarkers and identification of alternative treatment options. Increasing appreciation of the role of long non-coding RNAs (lncRNAs) in cancer progression has fostered efforts to characterize their role in disease biology and to evaluate them as novel biomarkers, as well as potential therapeutic targets [5–9].

LncRNAs are RNA transcripts that lack an open reading frame encoding a protein. LncRNAs are generally polyadenylated, greater than 200 bp in length and distinct from small RNAs and microRNAs [10–12]. In the past few years, lncRNAs have emerged as novel mechanisms in mediating cancer biology [13–18]. LncRNAs could act as an oncogene or tumor suppressor in tumor progression by affecting cell proliferation [19], differentiation [20], migration [15], immune response [13], and apoptosis [21]. A variety of mechanisms are involved in these tumor biological process such as remodeling of chromatin (*HOTAIR*, *XIST*, *ANRIL*) [22–24], transcriptional co-activation or co-repression (*H19*, *LincRNA-p21*) [21, 25], protein inhibition (*TERRA*) [26] post-transcriptional modifiers (*MALT1*) [27] or decoy elements (*PTENP1*) [28].

Genome-wide analyses indicate that IncRNAs frequently demonstrate restricted tissue-specific and cancer-specific expression patterns, distinguishing them from most miRNAs and protein-coding mRNAs [11, 29]. Moreover, lncRNA expression may confer clinical information about patient outcomes and have utility in diagnostic tests [22, 30, 31]. For example, prostate cancer gene 3 (PCA3) has been used as a biomarker in clinical practice for predicting prostate cancer volume [32]. *HULC* is reportedly detected in peripheral blood cells of HCC patients [33]. *HOTAIR* might be a biomarker for lymph node metastasis in HCC [34]. lncRNAs may also represent good candidates as therapeutic targets [35, 36]. *HOTAIR* can reduce the sensitivity of lung adenocarcinoma cells to chemotherapeutic drugs such as cisplatin [36]. Down regulation of *MALAT1* expression reduced tumor growth *in vivo* [37]. The characterization of the RNA species, their function, and their clinical applicability has therefore become an area of biological and clinical importance in cancer research.

High-throughput RNA sequencing (RNA-Seq) in human cancer shows remarkable potential to identify both novel markers of disease and uncharacterized aspects of tumor biology, particularly lncRNA species [12, 29]. We analyzed the RNA-Seq data on a large cohort of lung cancer tissues and cells lines to discover lncRNAs with diagnostic or prognostic use in lung cancer. We identified 281 differently expressed lncRNAs in LUAD and present our results from an in depth characterization of our top candidate *LINC00857*. We validated the differential expression in multiple independent datasets and established the prognostic importance of *LINC00857* expression. We next generated both cell line and xenograft models representing knockdown and overexpression of *LINC00857* to delineate its functions.

## RESULTS

### Differentially expressed lung lncRNAs discovery and cross-validation

We recently performed RNA-Seq on a large cohort of lung cancer samples [4] (UM cohort) including 113 lung cancer tissues (67 LUADs, 36 SCCs and 10 large cell lung cancers), 6 matched normal lung tissues, and 26 lung cancer cell lines (Supplementary Figure S1A, Supplementary Table S1). In that study we also compiled two large RNA-Seq datasets then available, to perform a comprehensive gene fusion analysis. In the current study we perform a comprehensive analysis on the gene expression data-matrix from these three cohorts to discover differentially expressed lncRNAs in LUAD. The three cohorts are the UM cohort described above and two large publically available RNA-Seq data namely the Korean cohort (Seo) [38] including 85 LUADs and 77 normal, and finally The Cancer Genome Atlas (TCGA) LUAD data [39] including 309 LUADs and 73 normal lung samples (Supplementary Figure S2). Mate-pair reads were aligned using TopHat against the Ensembl GRCh37 human genome and initial transcripts elucidated with Cufflinks. Expression levels of transcripts were represented as Fragments Per Kilobase, Per Million mapped reads (FPKM). A total of 55,400 transcripts were mapped and classified as protein-coding genes, pseudogenes, lncRNAs, etc. according to their overlap with known transcripts in the Ensembl 66 database.

**Figure 1:**
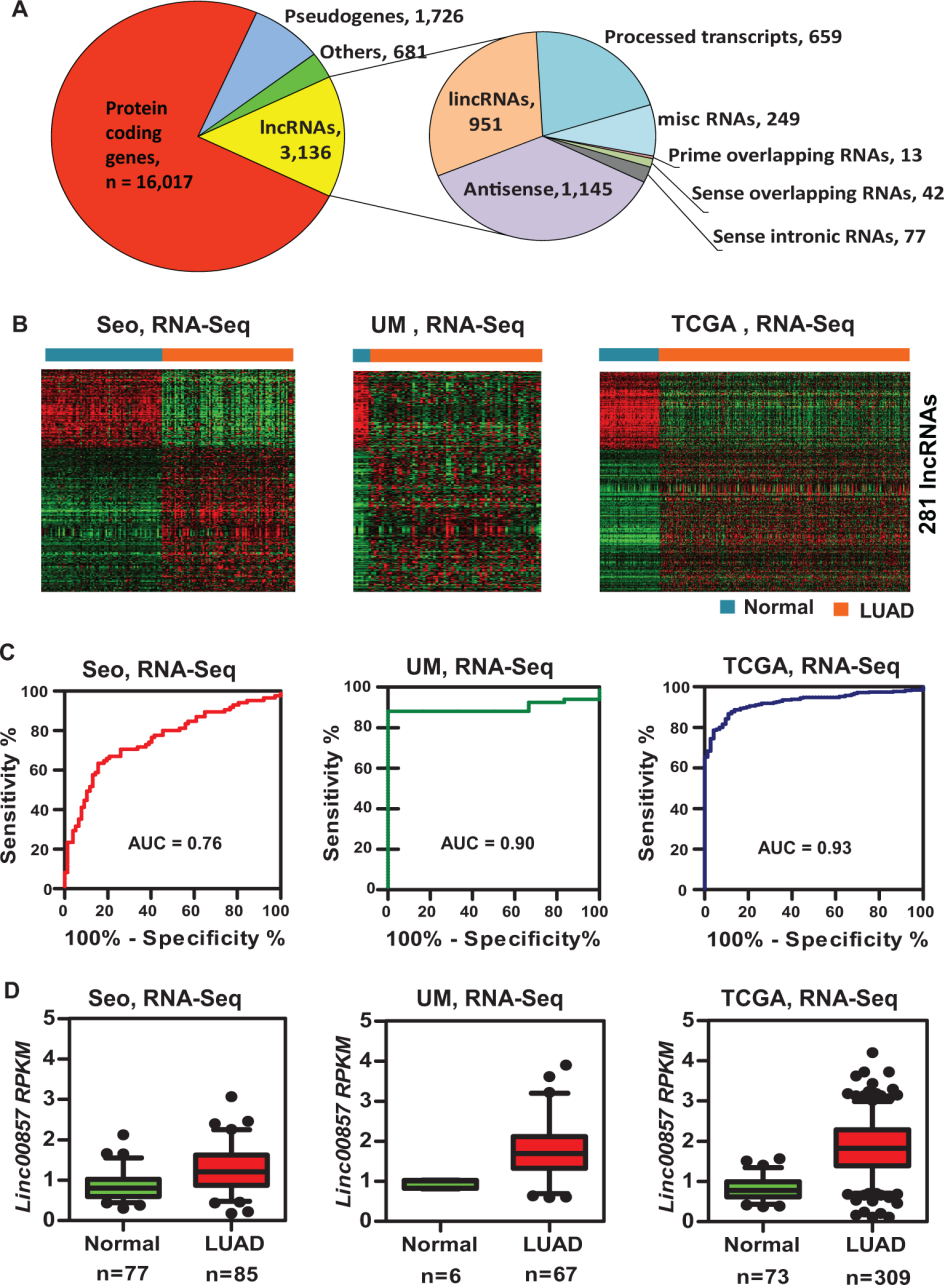
Differentially-expressed lncRNAs including *LINC00857* in lung AD and normal lung tissues (**A**) Classification of all transcripts observed in this study from RNA-Seq data on 119 lung samples. The left pie chart shows transcript distribution in lung cancer. LncRNA accounts for 3,136 of all 21,560 expressed transcripts. The right pie chart shows further subclassification of 3,136 lncRNAs. Antisense, lncRNAs and processed transcripts are the major type of lncRNAs expressed in lung cancer. (**B**) Heat maps showing 281 different expressed lncRNAs from UM (67 Ad vs 6 N), Seo (85 AD vs 77 N) and TCGA (309 AD vs 73 N) RNA-Seq data sets. Columns represent samples, and rows lncRNAs, red is high expression and green low expression. (**C**) ROC curves of *LINC00857* in Seo (85 AD vs 77 N), UM (67 AD vs 6 N) and TCGA (309 AD vs 73 N) RNA-Seq data sets. (**D**) Boxplots of *LINC00857* expression levels in AD and normal tissue samples in Seo, UM and TCGA RNA-Seq datasets (AD vs. N, *p* < 0.001 in all 3 data sets).

In order to find transcripts having higher expression value in lung tissues, we filtered the dataset using the following criteria; transcript FPKM value > 0 in at least 4 samples and a minimum of one sample with value > 4 among the 119 UM lung tissue samples (113 cancers and 6 normal lung tissues). Filtering excluded 33,480 genes from further analysis and indicated that a significant portion of the transcriptome has either very low to no expression in lung tissues. The remaining 21, 560 Ensembl genes belonged to various classes that include 16,017 protein-coding genes (74%), 1,726 pseudogenes (8%), and 3,136 lncRNAs (15%) (Figure 1A, left). The 3,136 lncRNAs include: 1,145 anti-sense, 951 lncRNAs, and 659 processed transcripts (Figure 1A, right). We found that protein coding genes had a higher expression level than non-coding RNAs (Supplementary Figure S1B).

Our analysis primarily aimed at identifying lncRNAs whose expression pattern had significant clinical utility. Towards this we developed a methodology to analyze existing high throughput RNA sequencing data, accounting for robust differential expression in cancer samples, stringent cross validation across multiple independent datasets/platforms and finally significant association with prognostic index (Supplementary Figure S2). Accordingly we performed Receiver Operating Characteristic (ROC) curve analysis, and the area under the curve (AUC) values was used to select the top list of differently expressed lncRNAs in LUAD. There were a total of 281 lncRNAs which had an AUC value larger than 0.7 (182 lncRNAs) or less than 0.25 (99 lncRNAs) in all 3 data sets (Figure 1B, Supplementary Figure S3A and S3B and Supplementary Table S2). Several known lung cancer-associated lncRNAs, such as *HOTAIR, GAS5, PVT1,* and *UCA1* [40] were found in this top list (Table S2, Figure S3C) which further supported the power of this analysis. Importantly 34 of these lncRNAs correlated to patient survival (Cox model) in lung cancer (Supplementary Table S3). We also observed that most of these lncRNAs were deregulated in large cell or squamous cell lung cancer (Supplementary Figure S4). These lncRNAs demonstrated unique expression patterns and suggested potential novel diagnostic or prognostic markers in lung cancer.

### *LINC00857* is overexpressed in lung AD and predicts poor patient survival

**Figure 2:**
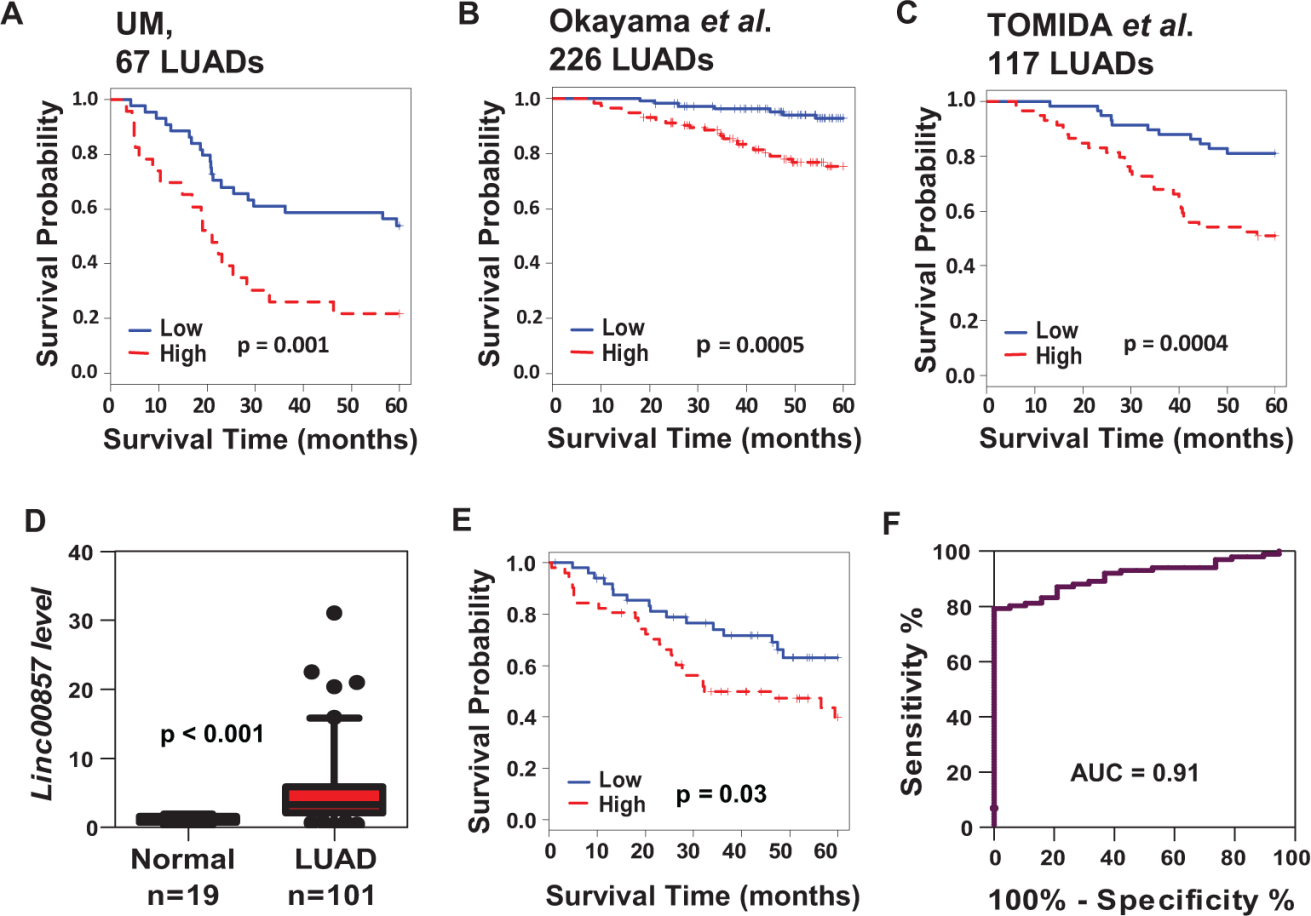
*LINC00857* was increased and significantly associated with poor patient survival in lung AD (**A**–**C**) Kaplan-Meier curves and log-rank test of *LINC00857* in UM (67 ADs), Okayama (226 ADs) and TOMIDA (117ADs) data sets indicating higher *LINC00857* expression was associated to poorer patient survival. (**D–F**) qRT-PCR validation of *LINC00857* expression in an independent data set including 19 normal and 101AD tissue samples. Boxplot (D) indicated *LINC00857* expression was increased in tumor (vs. normal). Kaplan-Meier curve (E) indicated higher *LINC00857* expression was unfavorable for patient survival, and ROC curve (F) indicated an excellent (AUC = 0.91) of classifying the 101 tumors from 19 normal based on *LINC00857* expression.

Among the most overexpresssed lncRNAs, *LINC00857* was found to be significantly increased in LUADs (Figure 1C, 1D and Supplementary Figure S5) and was associated with worse survival (*p* < 0.001) in our patient cohort (UM 67 LUADs) (Figure 2A). We next validated this association in two independent published LUAD microarray data sets where survival information was available namely, Okayama et al., *p* < 0.0005 (226 LUADs, stage 1 and 2) [41] and Tomida et al. *p* < 0.0004 (117 LUADs, stage 1 to 3) [42] (Figure 2B, 2C). To further confirm the diagnostic and prognostic significance of *LINC00857* in LUAD, we examined it's expression in an expanded UM cohort (101 lung ADs and 19 normal lung tissues) by an independent qRT-PCR assay. We found that *LINC00857* expression levels were significantly higher in LUAD as compared to normal lung tissues (Figure 2D) with an excellent performance (AUC = 0.91) for classifying the tumors from normal lung based on *LINC00857* expression (Figure 2F), and higher expression of *LINC00857* was significantly related to worse patient survival (Figure 2E). *LINC00857* expression levels and association with other clinical variables from this validation set are shown in Supplementary Table S3. In addition, we did not find any evidence for association between *LINC00857* expression and *KRAS* or *EGFR* mutation status in our analysis (data not shown).

To explore if *LINC00857* is expressed in other types of cancer, we downloaded 6,220 cancer RNA-Seq expression data from MiTranscriptome [29]. We found that higher expression (FRKM log2 value > 0.5) of *LINC00857* was presented in bladder, gastric, lung AD, cervix and pancreas cancers, and lower in prostate, thyroid, brain, blood and skin cancers (FRKM log2 value < 0.5) (Supplementary Figure S6A). When we compared to tumor vs. normal in 10 types of cancer which have normal tissues, we found that bladder, gastric, head & neck, liver, thyroid and lung cancers were significantly increased (vs. normal), whereas breast, kidney and prostate cancer were decreased (vs. normal) (Supplementary Figure S6B top panel). Surprisingly, we found that lung AD was the most significant one in term of *p* value by tumor vs. normal among these 10 types of cancer (Supplementary Figure S6B bottom panel). These indicated that *LINC00857* was highly expressed in some types of cancer especially in lung AD (vs. normal) and could be potential useful as lung AD specific marker.

In order to confirm the FPKM value of *LINC00857* detected by RNA-Seq and to select tumor cells for functional analyses, we measured *LINC00857* expression in 33 lung cancer cell lines using qRT-PCR. We found a strong correlation of *LINC00857* expression measured by RNA-Seq and qRT-PCR (Pearson *r* = 0.83, *p* < 0.001, Supplementary Figure S7). While, high *LINC00857* expression was observed in 29 of the 33 cell lines tested, it was not expressed in 3 non-adherent lung cancer cell lines including 2 small cell lung cancers (H82 and H526) and one NSCLC derived from a metastatic lymph node (H1155) showed no expression.

The bi-exonic *LINC00857* gene is located in the forward strand on chromosome 10q22.3 and is 2,171 bases long (Supplementary Figure S8A). To determine if *LINC00857* overexpression arises from genomic amplification, we examined potential DNA copy number alterations in 90 lung ADs by Affymetrix SNP6.0 array (unpublished data). Except for one tumor with genomic gain we did not find any aberrations in this region in any other sample (Supplementary Figure S8B). We also observed that knockdown of *LINC00857* using *LINC00857*-specific siRNA did not affect the expression of neighboring genes (Supplementary Figure S8C) indicating *LINC00857* expression is not involved in cis-regulation of its neighbors. Taken together, *LINC00857* is significantly overexpressed in LUAD across multiple studies and is a predictor of poor patient survival. Our data supports further examination of *LINC00857*'s clinical utility in larger multi-institutional cohorts with outcome data to examine whether this gene has diagnostic and/or prognostic importance for lung cancer.

### *LINC00857* promotes cancer cell proliferation and invasion

**Figure 3:**
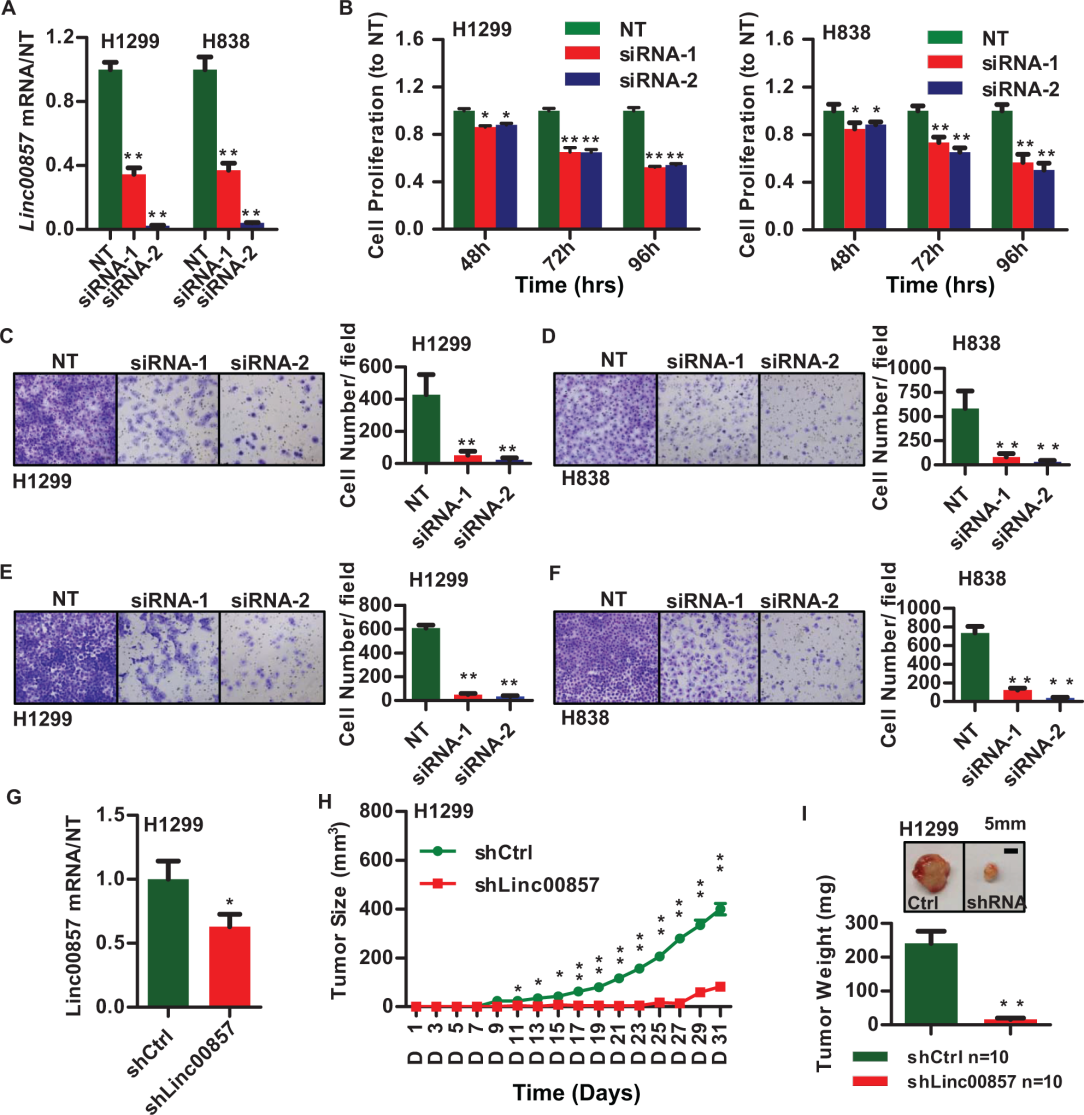
*LINC00857* is involved in tumor progression (**A**) *LINC00857* siRNA knockdown efficiency (48 h) in H1299 and H838 measured by qRT-PCR. (**B**) Cell proliferation decreased after *LINC00857* siRNAs treatment in H1299 and H838 cell lines. (**C–D**) *LINC00857* siRNA knockdown impaired cellular invasion ability in H1299 and H838 cell lines (10X). (**E–F**) *LINC00857* siRNA knockdown impaired cellular migration ability in H1299 and H838 cell lines (10 ×). (**G**) *LINC00857* shRNA knockdown efficiency in H1299 measured by qRT-PCR. (**H**) Mouse *in vivo* xenograft tumor growth curves of H1299 cells expressing control and *LINC00857* shRNA. (**I**) Tumor weight of H1299 cells expressing control and *LINC00857* shRNA. **p < 0.01, *n* = 10 mice/group.

To examine the functional role of *LINC00857*, we performed small interfering RNA (siRNA)-mediated knockdown in H1299 and H838, two lung LUAD cancer cells, which contains high levels of the endogenous *LINC00857* transcript (Supplementary Figure S7A and S9A). These two cell lines also exhibit high invasive potential among a panel of lung cell lines when assessed using the Boyden chamber assay (data not shown). To minimize the possibility of off-target effects, we employed two independent gene-specific siRNAs whose knockdown efficiency were 60 and 90% respectively, as estimated by qRT-PCR (Figure 3A). A significant decrease in cell proliferation in both H1299 and H838 cell lines (Figure 3B) and reduced cancer cell invasion (as measured by Boyden chamber matrigel invasion assays) was noted when *LINC00857* was knocked down (Figure 3C and 3D). Similarly *LINC00857* abrogation resulted in a significant loss in cell migration (Figure 3E and 3F) and colony formation (Supplementary Figure S9B).

Having demonstrated a role for *LINC00857* in cell growth and migration using the knockdown model, we next focused on probing the overexpression phenotype. Towards this end, we generated a full length *LINC00857* expression construct in a pcDNA-DEST53 vector and transfected the lung cancer cell line SK-LU-1, which possesses low endogenous levels of *LINC00857* as compared to H1299 and H838 (Supplementary Figure S9A). Contrary to knockdown experiments, overexpression of *LINC00857* promoted cancer cell colony formation (Supplementary Figure S9C and S9D), cell proliferation and invasion in SK-LU-1 cells (Figure S9E and S9F). Furthermore subcutaneous injections in nude mice (*n* = 10 mice/group) with *LINC00857* stable knock down H1299 cells showed impaired tumor growth (Figure 3G–3I). These results reaffirm our findings that *LINC00857* plays a critical role in lung cancer cell proliferation, migration, invasion and colony formation. Thus *LINC00857* may not only be a potential biomarker for diagnosis and prognosis of LUAD, but also play an important role in lung cancer progression.

We next looked at cellular localization of this lncRNA in H1299 and H838 cells. Quantitative RT-PCR of total RNA isolated from total, nuclear and cytoplasmic fraction of these two cell lines revealed enrichment in the cytoplasmic fraction (Supplementary Figure S10A). Interestingly, the A549 RNA-Seq data from the Encode database also shows cytoplasmic enrichment of *LINC00857* providing further support (Supplementary Figure S10B). Further identification of the *LINC00857* direct binding proteins using RNA pull-down assay or identifying the functional domains of *LINC00857* using domain-specific chromatin isolation by RNA purification (dChIRP) [43] are currently being investigated.

### *LINC00857* knockdown causes cell cycle arrest at G1/S via CCNE1 regulation

**Figure 4:**
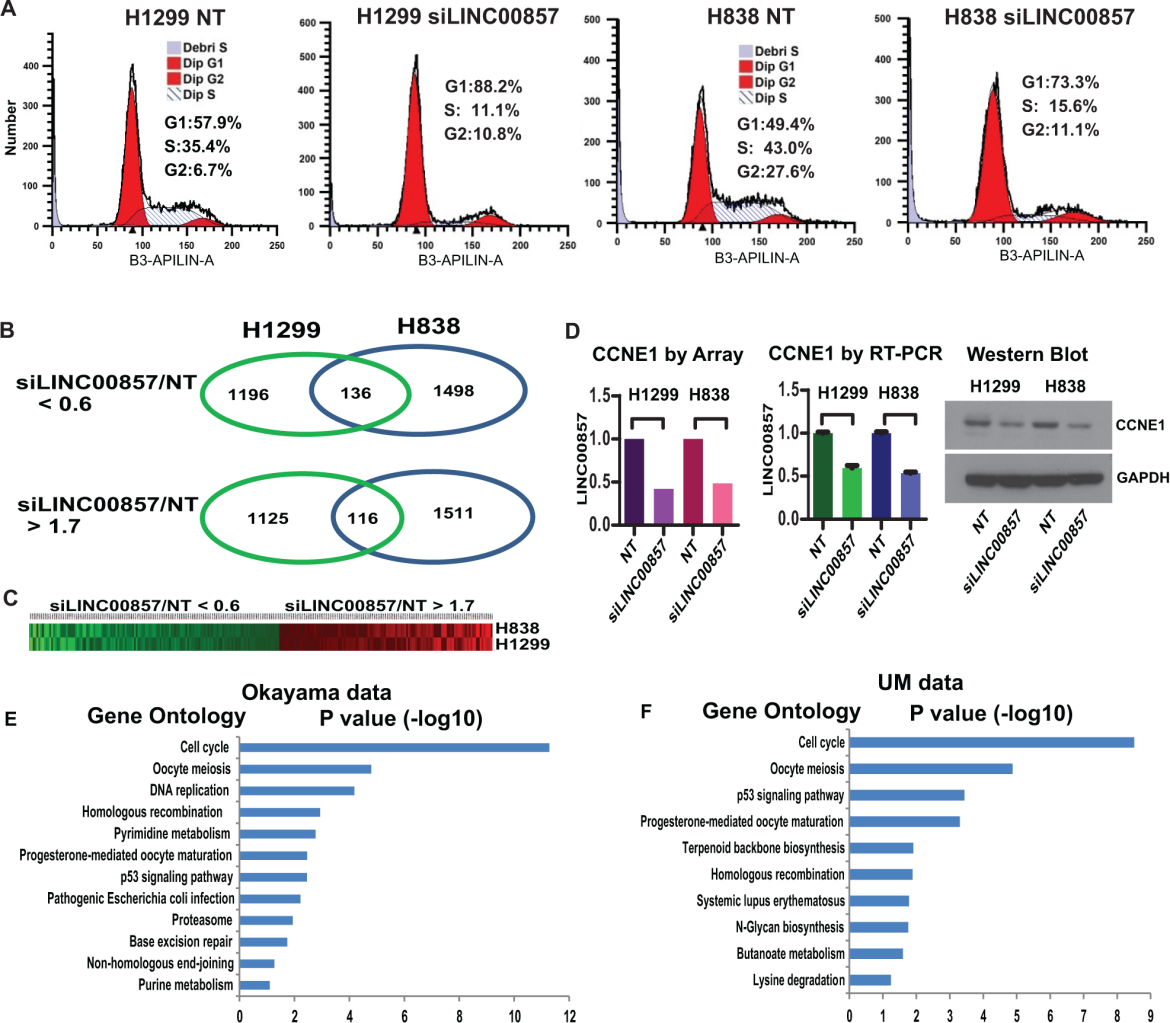
*LINC00857* regulates cell cycle at the G1/S phase and potentially through CCNE1 and CDK2 regulation (**A**) Knockdown using *LINC00857* siRNA caused G_1_ arrest in both H1299 and H838 lung cells. (**B–C**) *LINC00857* siRNA knockdown induced 136 down and 116 up-regulated genes in both H1299 and H838 cells. (**D**) *CCNE1* was decreased after LINC00857 siRNA treatment measured by both microarray and qRT-PCR for mRNA, and protein by Western blot. (**E–F**) The *LINC00857* positively correlated genes in primary tumors were significantly involved in cell cycle regulation analyzed by gene ontology from DAVID.

To help determine how *LINC00857* affects tumor growth, we utilized flow cytometry analysis to examine the effect of *LINC00857* knockdown on the cell cycle. As shown in Figure 4A, knockdown of *LINC00857* using siRNA, induced cell cycle arrest at the G_1_/S phase indicating that G_1_ arrest might be one mechanism for how *LINC00857* knockdown decreased lung tumor cell growth.

Next, in order to investigate potential genes specifically regulated by *LINC00857*, we performed whole transcriptome analysis using Affymetrix ST2.1 exon arrays after *LINC00857* siRNA knockdown in H1299 and H838 cell lines. As shown in Figure 4B and 4C, 1196 and 1498 genes were down-regulated at least 40% (relative to non-target siRNA control) in H1299 and H838 cells, respectively, and 136 genes were down-regulated in both cell lines (Supplementary Table S4) and we independently validated several dysregulated genes using qRT-PCR (Supplementary Figure S11A and S11B). The expression of cyclin E1 (CCNE1) (Figure 4D) and cyclin-dependent kinase 2 (CDK2) (Supplementary Figure S11C) were decreased after *LINC00857* knockdown supporting the premise that *LINC00857* may affect the cell cycle at G_1_/S through regulation of CCNE1 and CDK2 genes (Supplementary Figure S11D). Other cell cycle related genes regulated by *LINC00857* knockdown are listed in Supplementary Table S5.

Next, we performed DAVID Gene Ontology analysis on genes positively correlated (Pearson correlation) with *LINC00857* based on primary tumor data using Affymetrix microarrays [41] and UM RNA-Seq data [4]. Surprisingly, we found that *LINC00857* positively correlated genes including *CCNE1* and *CDK2* (Supplementary Table S6) in primary tumors were significantly involved in cell cycle regulation (Figure 4E and 4F, and Supplementary Figure S12) which further supports *LINC00857* involvement in cell cycle regulation.

There were 1125 and 1511 genes up-regulated at least 1.7-fold in H1299 and H838 cells, respectively, with 116 genes up-regulated in both cells (Figure 4B and 4C).

## DISCUSSION

To date, only a few lncRNAs (including *MALAT1, HOTAIR, H19, MEG3, GAS5, ANRIL,* and *SOX2OT* etc.) have been reported to be dysregulated and functionally characterized in lung cancer and most of the studies were based on microarray expression data [40, 44, 45]. In comparison whole transcriptome sequencing provides a more comprehensive quantitative assessment of expressed transcripts without any probe limitations (associated with microarray analysis) and allows identification of novel and important lncRNAs which may have diagnostic, prognostic and functional potential in lung cancer [12, 29, 46].

In this study, there were 56,369 transcripts that could be matched to the Ensembl database, but only 3,136 lncRNAs were expressed in lung tissues indicating the tissue specific expression pattern of lncRNAs (Figure 1A). Based on three large RNA-Seq data sets containing a total of 461 LUADs and 156 normal lung tissues, we have identified 281 lncRNAs which were differently expressed in LUADs vs normal with an AUC large than 0.7 (higher in tumor) or less than 0.25 (lower in tumor) in all three data sets (Supplementary Figures S2, S3, Figure 1B). Some of the lncRNAs were also related to patient survival in lung cancer although some survival related lncRNAs may have been missed due to the selection criterion using AUC (tumor vs normal) rather than survival analysis by the Cox model. These lncRNAs could potentially serve as diagnostic or prognostic biomarkers in lung cancer. One of the top dysregulated lncRNAs *LINC00857* was further validated for its diagnostic or prognostic potential using 3 independent data sets with different platforms including microarray and qRT-PCR (Figure 2).

Functional characterization indicated that *LINC00857* plays an important role in tumor proliferation, migration and invasion. We also found that *LINC00857* regulated the cell cycle by causing G_1_/S arrest through regulating CCNE1 and CDK2 expression. The *LINC00857* genomic locus was not amplified (Supplementary Figure S8B), thus we hypothesize that the cancer specific *LINC00857* expression may be mediated by specific transcription factors coupled with changes in histone and DNA modification (H3K27 acetylation) in its gene promoter. Preliminary *in silico* analysis of a 1.2 kb *LINC00857* promoter region (−1000 to +200 of transcription start site) using MatInspector tool, reveals the presence of DNA binding elements for transcription factors such as CREB, CTCF, NFKB and STAT (Supplementary Figure S13A and S13C). The existing ENCODE data on DNA methylation and transcription factor binding/histone marks from Chip-Seq includes the lung cancer cell line A549 (which has high levels of *LINC00857* expression). The ENCODE Infinium 450K methylation data reveals that the *LINC00857* promoter is unmethylated in A549 (compared to prostate cell line LNCaP where it is fully methylated). While the Chip-Seq data suggests binding of CTCF and CREB (these binding elements were noted in our *in silico* analysis of *LINC00857* promoter) along with increased histone acetylation marks in A549 cells (Supplementary Figure S13B). The insights gained from these observations will help inform future detailed *LINC000857* promoter characterization by generating promoter-reporter constructs (with luciferase as the reporter gene) of various lengths and studying their activity by transfecting them into lung cancer and normal cell lines. This will help us narrow down the critical regions in the promoter and potential transcription factors that regulate *LINC000857* expression. In addition we will also look at both DNA and histone modifications of this promoter region in cell lines that exhibit varying levels of *LINC00857* expression.

Taken together, our study provides a comprehensive analysis of newly characterized *LINC00857* in lung cancer. It establishes the role of *LINC00857* as potential driver of lung cancer pathogenesis and a diagnostic or prognostic biomarker of disease.

## MATERIALS AND METHODS

### Cell lines

H1299, H838, SK-LU-1 cells were maintained in RPMI 1640 or EMEM supplemented with 10% FBS and 1% antibiotic-antimycotic. All cell lines were cultured at 37°C in a 5% CO2 cell culture incubator. All cell lines were obtained from the American Type Culture Collection and maintained using standard media and conditions. All cell lines were genotyped for identity at the University of Michigan Sequencing Core and were tested routinely for Mycoplasma contamination.

### Tissue samples

The lung cancer and paired non-tumor lung tissues were obtained from patients undergoing curative cancer surgery during the period from 1991 to 2012 at the University of Michigan Health System. None of the patients included in this study received any preoperative radiation or chemotherapy. All the patients provided informed consent. This project received approval from the University of Michigan Institutional Review Board and Ethics Committee. Resected specimens were frozen in liquid nitrogen and then stored at −80°C until use. Another portion of the tissues were fixed in 10% formalin and embedded in paraffin for histopathological analysis. Frozen tissues for regions containing a minimum of 70% tumor cellularity defined by cryostat sectioning, were utilized for RNA isolation. The median follow-up time was 8.12 years among the patients that remained alive. The clinical information of LUAD samples used in RNA-Seq (67 LUADs) and qRT-PCR validation (101 LUADs) was in Supplementary Tables S1 and S3.

### RNA isolation, RNA sequencing and quantitative RT-PCR

Total RNA was isolated from tissue samples and cell lines followed by column purification using miRNeasy Mini-kit (Qiagen) according to the manufacturers' instructions. RNA quality was analyzed by 2100 Bioanalyzer. Samples with RNA integrity number (RIN) > 8.0 were subjected to RNA-Seq. Polyadenylated mRNAs were used to generate strand specific, bar-coded, paired-end Illumina sequencing libraries and sequenced on an Illumina Hi-Seq 2000. cDNA was prepared from RNA samples using High Capacity cDNA Reverse Transcription kit (Applied Biosytems) according to manufacturer's instructions. Quantitative real-time reverse transcription polymerase chain reaction (qRT-PCR) was prepared using Power SYBR Green master Mix (Life Technology Inc.) and was performed with an ABI StepOne Real-Time PCR System (Applied Biosystems). Each sample had a final volume of 15 μL containing approximately 20 ng of cDNA. The oligonucleotide primers for *LINC00857* were in Supplementary Table S7. The housekeeping genes *GAPDH* and *ACTB* were used as loading controls. Fold-changes were calculated relative to housekeeping genes and were normalized to the median value in normal samples.

### RNA sequencing data analysis

Mate-pair reads were aligned using TopHat [47] against the Ensembl GRCh37 human genome and initial transcripts elucidated with Cufflinks [47, 48]. Expression levels of transcripts were represented as Fragments Per Kilobase Per Million mapped reads (FPKM) [11, 47]. Transcripts were then classified as protein-coding genes, pseudogenes, lncRNAs, etc. according to their overlap with transcripts in known Ensembl database. There were 56,369 transcripts which could be matched to the Ensembl database with many having very low or no expression value. Because a 250–300bp size selection was employed in the RNA-Seq library generation protocol, transcripts that are less than 250bp in length (such as miRNA, snoRNA or snRNA), with background noise or low-level expressed transcripts were removed from further analysis [11].

### Published microarray and RNA sequencing data collections

Two published microarray data sets representing 343 primary lung AD tissues were utilized. These included Okayama et al., 226 LUADs with stage 1 and 2 [41], and Tomida et al., with 117 stage 1 to 3 LUADs [42]. The CEL files of microarray data were normalized using Robust Multi-array Average (RMA) method [49]. We also obtained Seo and TCGA RNA-Seq data sets [38, 39] consisting of a total of 394 ADs, 212 SCCs and 150 normal lung tissues. Expression levels of transcripts were represented as FPKM [47]. Our primary outcome was overall survival, censored at five years. The information concerning adjuvant chemotherapy or radiation therapy was provided in the original papers.

### Immunoblot analysis

For immunoblot analysis (Western blot analysis), lung cancer cell lysates were boiled in sample buffer, separated by polyacrylamide gel electrophoresis, and transferred onto polyvinylidene difluoride membranes. After blocking for 1 h with 5% non-fat milk, the membranes were incubated with primary monoclonal antibodies against human CCNE1 (1:1000 dilution), GAPDH (1:10,000 dilution) overnight at 4°C. After incubation with HRP-conjugated secondary antibody (at a 1:2000 dilution) for 1 h at room temperature, the membranes were developed using ECL and exposed to X-ray film.

### siRNA-mediated knockdown

Cells were plated in 60 mm plates at a desired concentration and transfected with 10 nM experimental siRNA oligonucleotides or non-targeting controls 24 h after plating. Knockdown was performed with Lipofectamine^®^ RNAiMax Reagent (Invitrogen, USA) in OptiMEM medium according to the manufacturer's instructions. Knockdown efficiency was determined by qPCR. *LINC00857* siRNA sequences for knockdown experiments are provided in Supplementary Table S8.

### Overexpression

Full-length *LINC00857* transcript was bought from Thermo and cloned into the Gateway pcDNA-DEST53 vector (Invitrogen) along with control sequence according to the manufacturer's instructions. Insert sequences were confirmed by Sanger sequencing at the University of Michigan Sequencing Core. The lung cancer cell lines SK-LU-1 with low expression of *LINC00857* was used and isolated cells generated by selection with Geneticin (Gibco).

### Cell proliferation assay

Cells were plated in 96 well plates at a desired concentration and transfected with 10 nM experimental siRNA oligonucleotides or non-targeting controls at 24 h after plating. Knockdown was performed with Lipofectamine^®^ RNAiMax Reagent in OptiMEM medium. At 48 h, 72 h, 96 h after transfection with siRNA, the proliferation rates were measured by Cell Proliferation Reagent (WST-1) (Roche) according to manufacturer's instructions. The cell viability percentages were calculated by normalizing to the survival fraction of the non-target siRNA group.

### Basement membrane matrix invasion assays

For invasion assays, cells were treated with the indicated siRNAs. After 48 h transfection, cells were trypsinized, counted with a Coulter counter and diluted to a desired concentration (H1299: 0.5 × 10^5^; H838: 1 × 10^5^. 0.5 ml cell suspension per well). Cells were seeded onto basement membrane matrix Boyden chambers (8-mm pore size, BD) present in the insert of a 24-well culture plate (Matrigel was purchased from BD Company). 20% FBS was added to the lower chamber as a chemoattractant. After 48 h, the non-invading cells and EC matrix were gently removed with a cotton swab. Invasive cells located on the lower side of the chamber were stained with Diff-QuikTM Stain Set (SIEMENS), air dried and photographed.

### mRNA expression detection using gene expression array

After siRNA treatment for 48 h on H1299 and H838 cells, the RNAs were collected for gene expression array. Non-target siRNA was used as the control group. Affymetrix Human Gene ST2.1 exon arrays were utilized to detect the RNA expression processed in the University of Michigan Genomics Core.

### Cell cycle analysis by flow cytometry

Forty-eight hours after treatment with *LINC00857* siRNA and/or control siRNA, H1299 and H838 cells were collected and then fixed with 70% ice-cold ethanol, washed with PBS, re-suspended in 1 ml of propidium iodide (PI) staining solution (0.1% (v/v) Triton X-100, 10 μg/mL PI, and 100 μg/mL DNase-free RNase A in PBS), and then incubated for 30 min at room temperature in the dark. Samples were transfered to the flow cytometer and used to measure the cell cycle in University of Michigan flow cytometry core.

### shRNA-mediated stable knockdown

The lung cancer cell line H1299 was seeded at 50–60% confluency and allowed to attach overnight. Cells were transfected with pTRIPZ *LINC00857* shRNA or with non-targeting shRNA using Thermo Scientific^™^ TurboFect^™^ *in vitro* Transfection Reagent for 48 h. RFP-positive cells were selected with 1.5 μg/ml puromycin for 1 week. At 48 h after the start of selection, cells were collected for both protein and RNA using RIPA buffer or TRIzol, respectively, as described above.

### Mouse xenograft model

All experimental procedures were approved by the University Committee for the Use and Care of Animals (UCUCA). To evaluate the role of *LINC00857* in tumor formation and growth, we propagated stable *LINC00857* knockdown H1299 pools using *LINC00857* shRNAs and non-targeting shRNA control cells and inoculated 1 × 10^6^ cells into the dorsal flank of 5–7 week-old nude mice (CB-17 SCID), using a Matrigel scaffold (BD Matrigel Matrix, BD Biosciences) (*n* = 10 mice/group). Tumor size was measured every 2 days, and tumor volumes were calculated using the formula (π/6) (L × W^2^), where L = length of tumor and W = width and represented as mm^3^/tumor. Post-monitoring, tumors were dissected from the animals, photographed and weighed. Tumor weight is represented by mg/tumor. Drinking water containing 2 mg/ml Doxycycline [50] was changed every two days.

### Statistical analyses for experimental studies

Data were analyzed using GraphPad Prism 6 (GraphPad software) and R software. To select the top list of differently expressed lncRNAs in LUAD (vs. normal), we performed Receiver Operating Characteristic (ROC) curve analysis. It showed the tradeoff between sensitivity and specificity (any increase in sensitivity will be accompanied by a decrease in specificity) for the different possible cut-points of a diagnostic test. The diagnostic accuracy was measured by the area under the curve (AUC). An AUC of 1 represented a perfect test; an AUC of 0.5 represented an imprecise test. Survival curves were plotted using the Kaplan-Meier method and survival differences were assessed by the log-rank test using the median of *LINC00857* as cutoff value. Univariate or multivariate (adjusted by age, gender, stage and differentiation) Cox proportional hazards models were calculated considering lncRNAs as a continuous variable. To identify pathway/gene expression patterns, an unsupervised hierarchical cluster analysis using un-centered average linkage was performed using Cluster v3.0 after mean-centering genes and arrays and heat maps were visualized using Tree View software [51]. The other data such as proliferation were evaluated by unpaired Student's *t*-test. A two-tailed *p* value < 0.05 was considered significant. To determine potential underlying biological processes associated with *LINC00857* correlated or regulated genes, Gene Ontology enrichment analysis was performed based on significantly correlated genes using DAVID bioinformatics website [52].

## SUPPLEMENTARY MATERIALS FIGURES AND TABLES


