# Supplementary material for: Non-coding RNA LINC00857 is predictive of poor patient survival and promotes tumor progression via cell cycle regulation in lung cancer
Source: Oncotarget. 2016 Feb 5;7(10):11487–99. doi: 10.18632/oncotarget.7203 (PMC4905488; doi:10.18632/oncotarget.7203)
Supplement: Supplementary file 1 [file oncotarget-07-11487-s001.pdf]

Non-coding RNA *LINC00857* is predictive of poor patient survival and promotes tumor progression via cell cycle regulation in lung cancer

Supplementary Materials

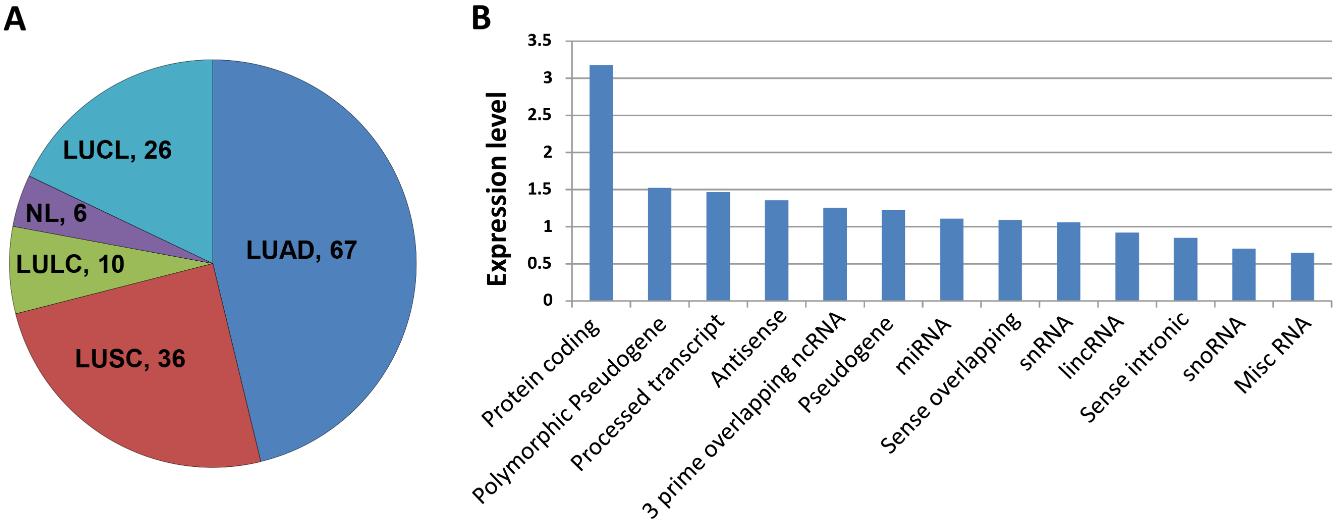

Supplementary Figure SI: (A) Samples composition of the university of michigan RNA-Seq cohort in this study. LUAD: lung adenocarcinoma, LUSC: lung squamous cell, LULC: lung large cell, NL: normal lung, LUCL: lung cell line. (**B**) The comparison of the RNA expression between protein coding genes and non-coding RNAs. Log 2 FPKM value.

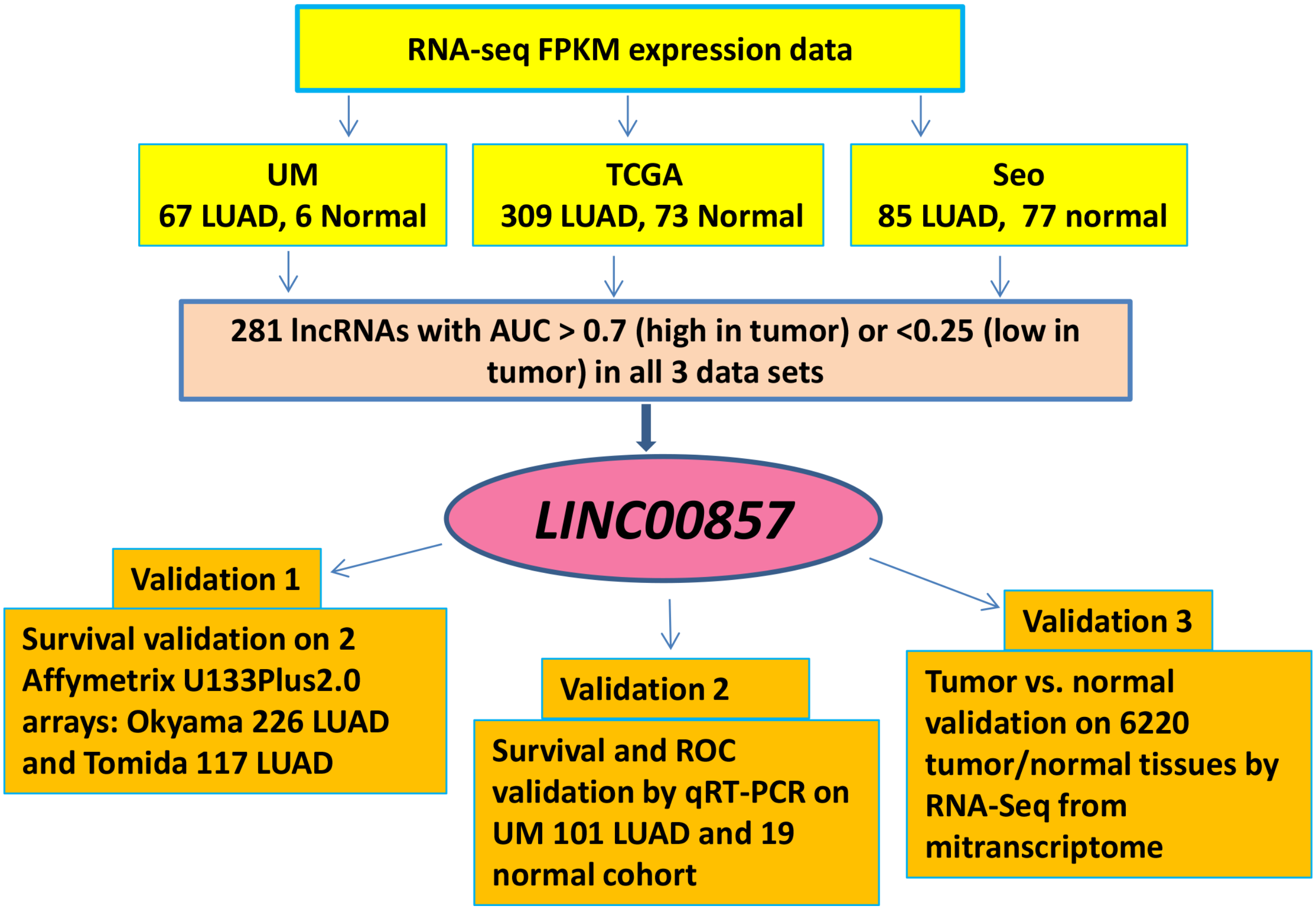

Supplementary Figure S2: A schematic flow chart of the discovery and validation of *LINC00857* in lung cancer.

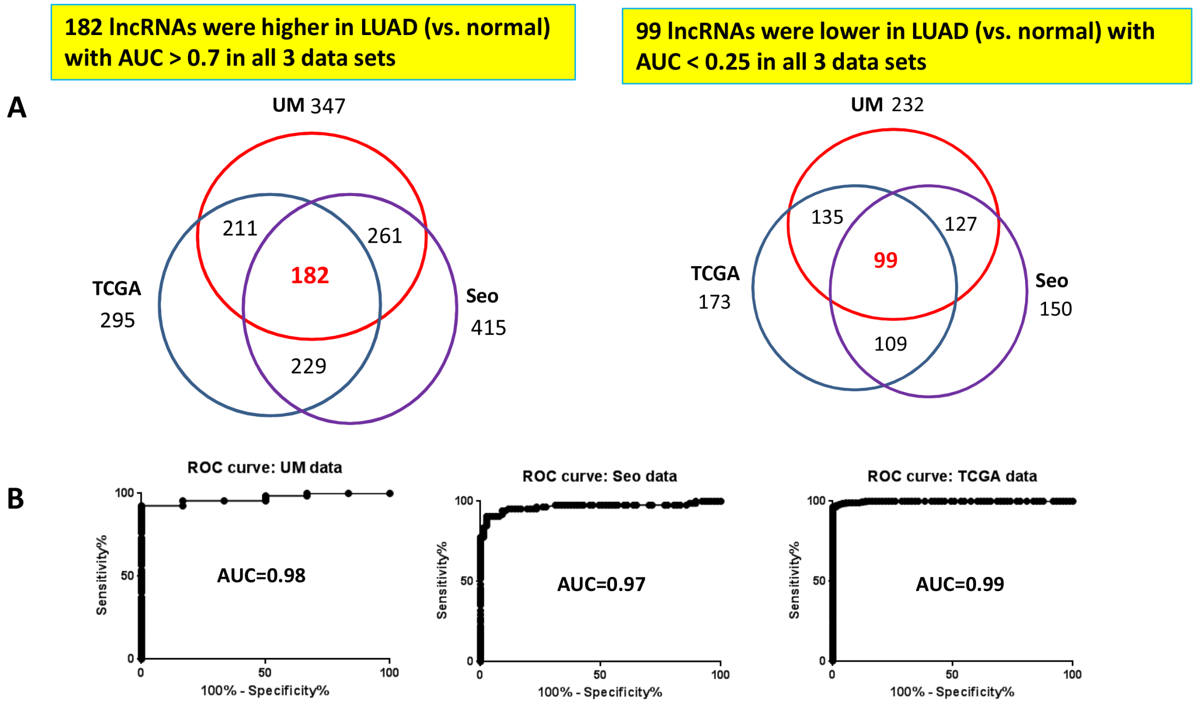

Supplementary Figure S3: (A) Selection of 281 IncRNAs based on AUC on all 3 RNA-Seq data sets. Number of IncRNAs in each group is indicated (**B**) Representative ROC curves of one IncRNA in 3 data sets.

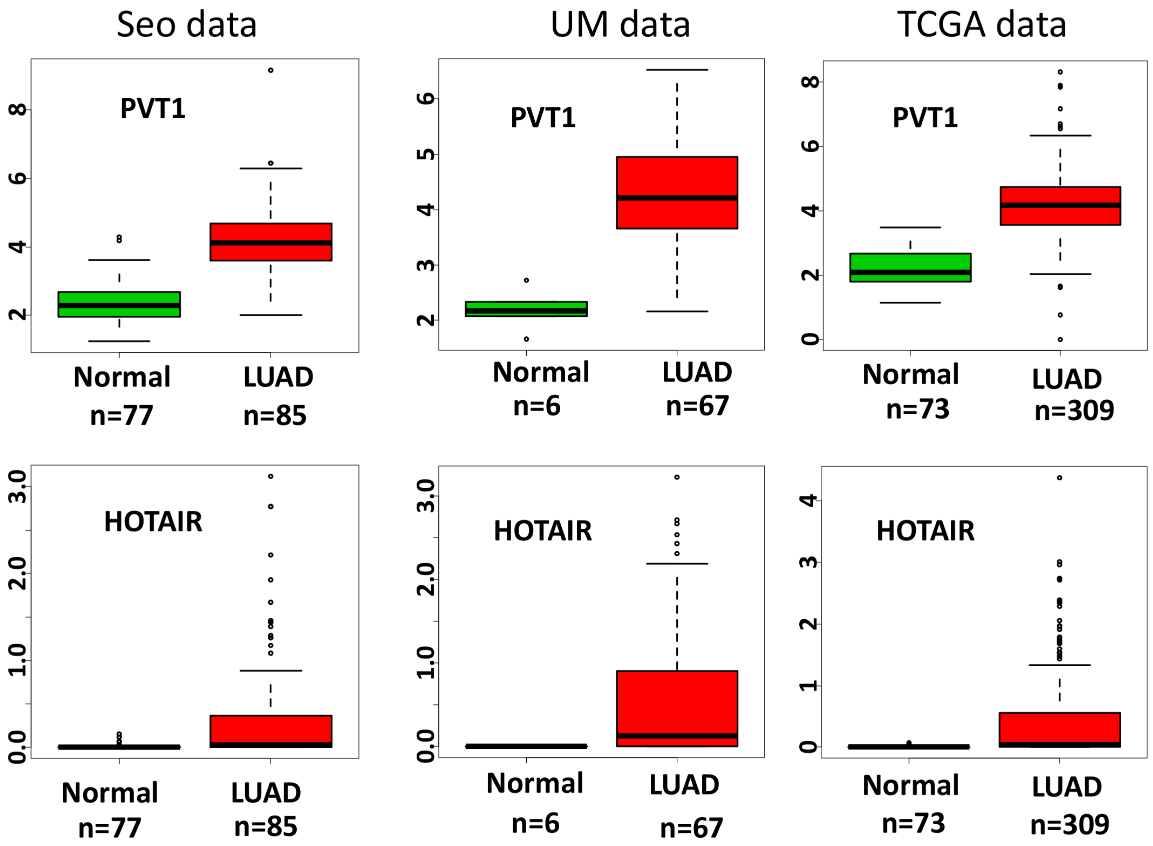

Supplementary Figure S3C: Representative of boxplot for two known lung cancer-associated IncRNAs, PVT1 and HOTAIR, in 3 RNA Seq data sets (p < 0.001).

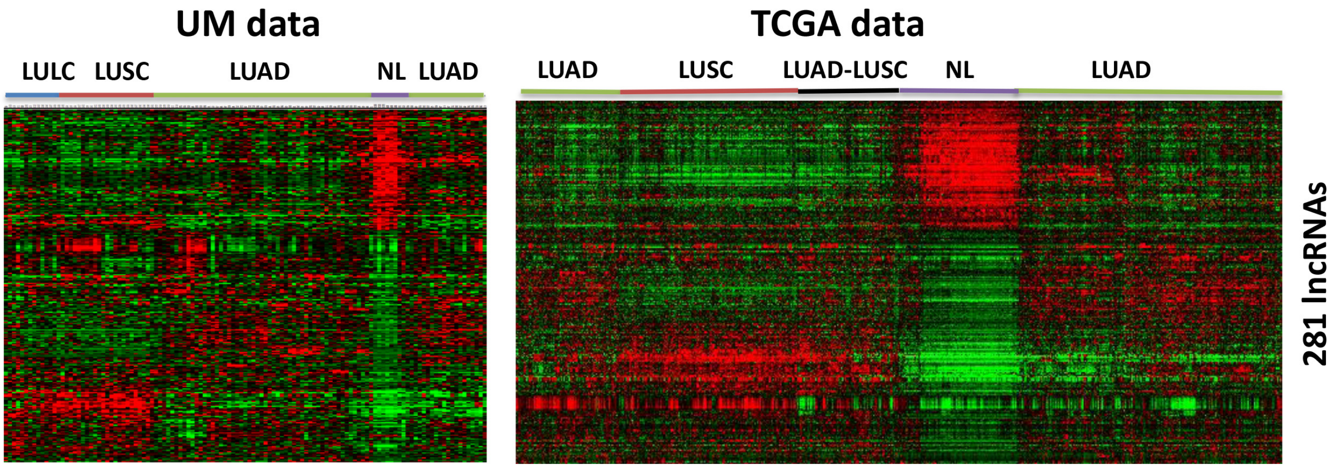

Supplementary Figure S4: (left panel) Expression pattern of 281 IncRNAs in UM 119 samples including 10 large cell lung cancers (LULC), 36 squamous cell lung cancers (LUSC), 67 lung adenocarcinomas (LUAD) and 6 normal lung tissues (NL). (right panel) Expression pattern of 281 IncRNAs in TCGA samples including 212 squamous cell lung cancers (SCC), 309 lung adenocarcinomas (AD) and 73 normal lung tissues (**N**).

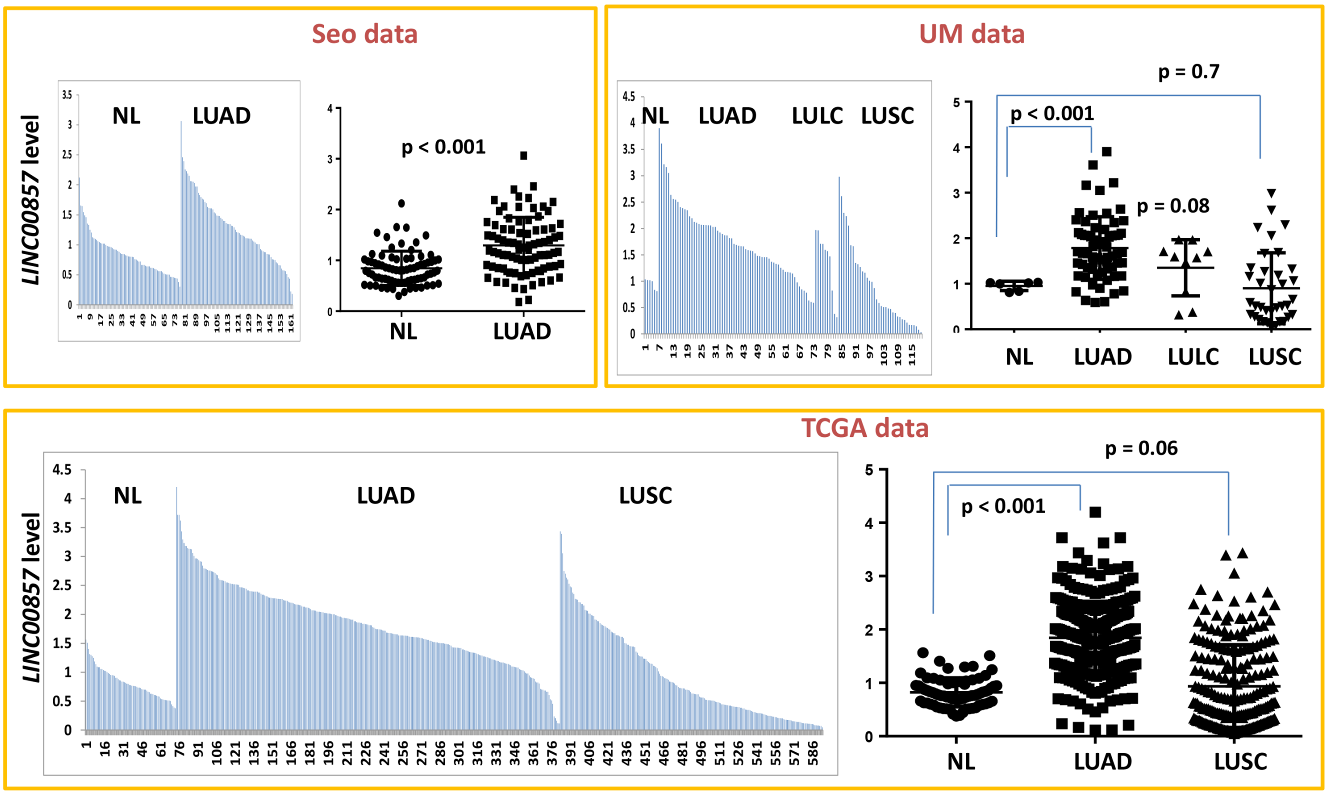

Supplementary Figure S5: Column and scatter plots of *LINC00857* expression level in all samples measured by RNA-seq (RPKM, log2) normal lung tissue, AD: adenocarcinoma, LC: large cell lung cancer, SCC: squamous cell lung cancer.

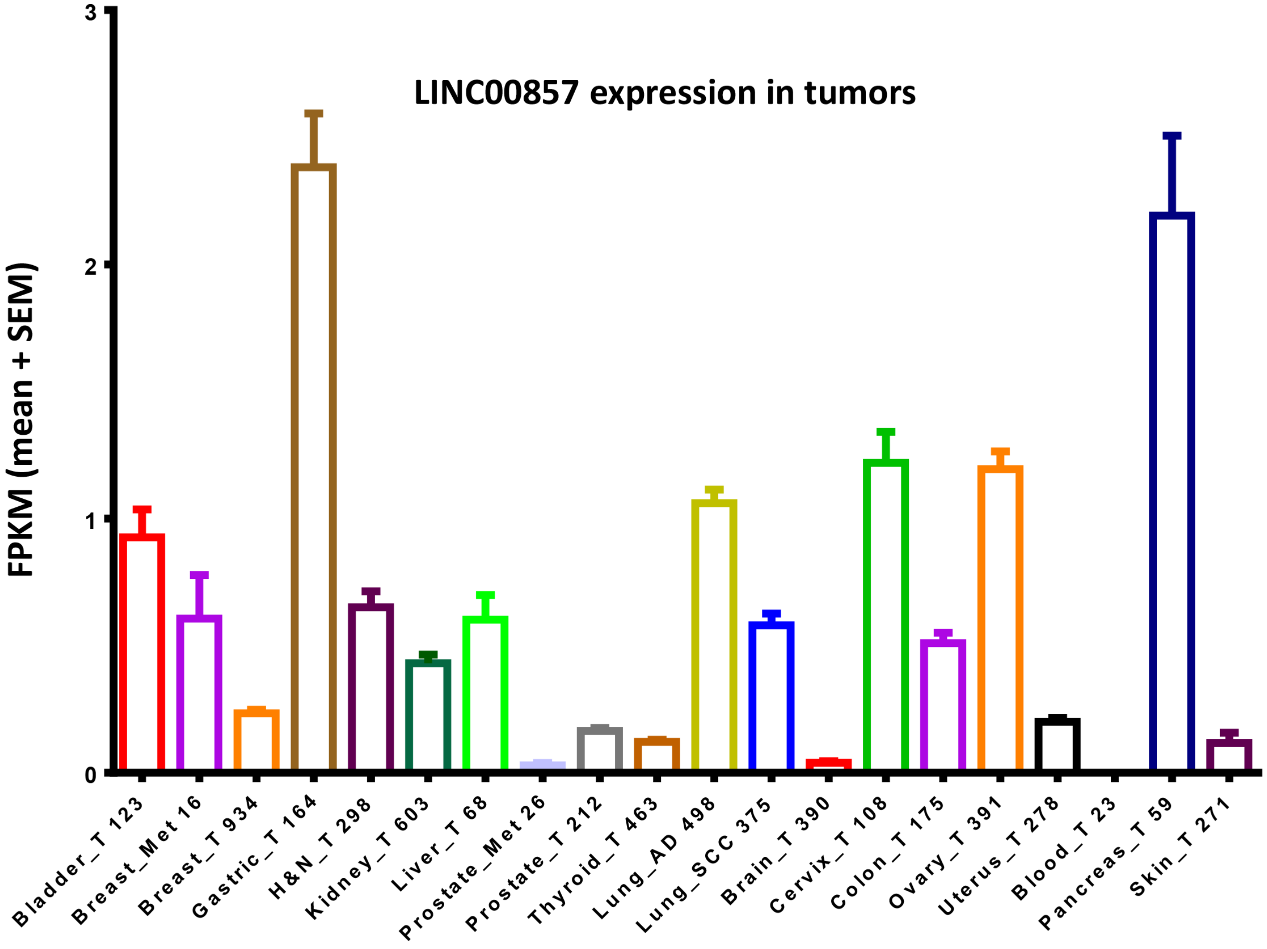

Supplementary Figure S6A: *LINC00857* expression in different type of cancer (mean + SEM, FPKM log2 value). High expression of *LINC00857* (FRKM log2 value > 0.5) were found in most type of cancers except prostate, thyroid, brain, blood and skin cancer (FRKM < 0.5). H & N, head and neck; AD, adenocarcinomas; SCC squamous cell cancer; case number of each cancer is indicated after T. The original data was download from http://mitranscriptome.org, with modification.

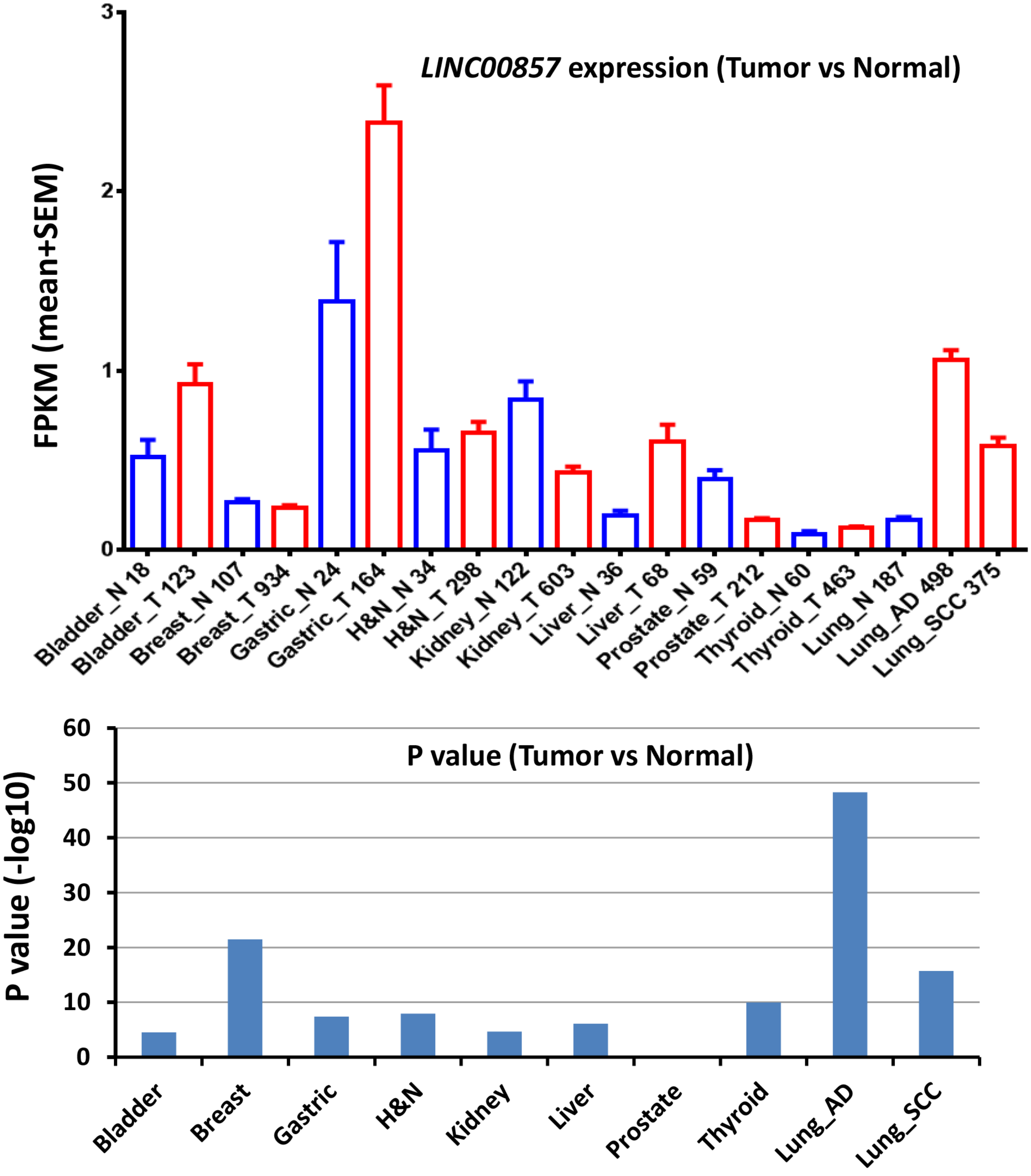

Supplementary Figure S6B: (Up panel), comparison of *LINC00857* expression in cancers (red) and it's normal (blue) tissues (mean + SEM, FPKM, log2). Bladder, gastric, head & neck, liver, thyroid and lung cancer are significantly increased, whereas breast, kidney and prostate cancer are decreased (vs. normal). Case number of each cancer is indicated. The original data was downloaded from mitranscriptome, with modification. (Bottom panel), *p* value (–loglO) of tumor vs. normal for each type of cancer in A. Lung AD is the most significant one in term of *p* value (vs. normal, *t* test).

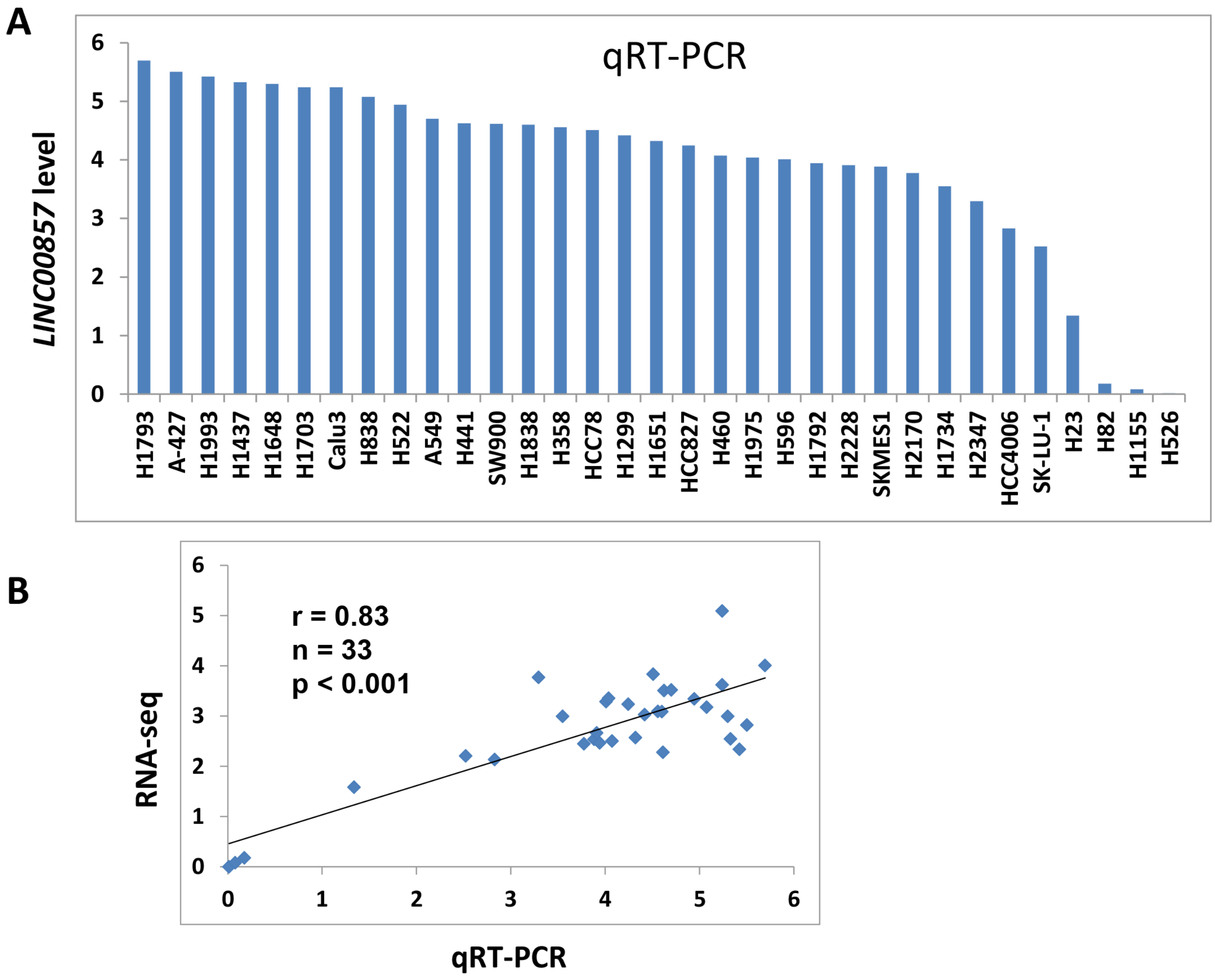

Supplementary Figure S7: *LINC00857* expression in 33 lung cancer cell lines. (**A**) *LINC00857* expression in 33 lung cell lines measured by qRT-PCR (log2 of relative to mean value). (**B**) Significantly positive correlation of *LINC00857* level measured by RNA-Seq (log2 of FPKM value) and RT-PCR based on 33 lung cancer cell lines.

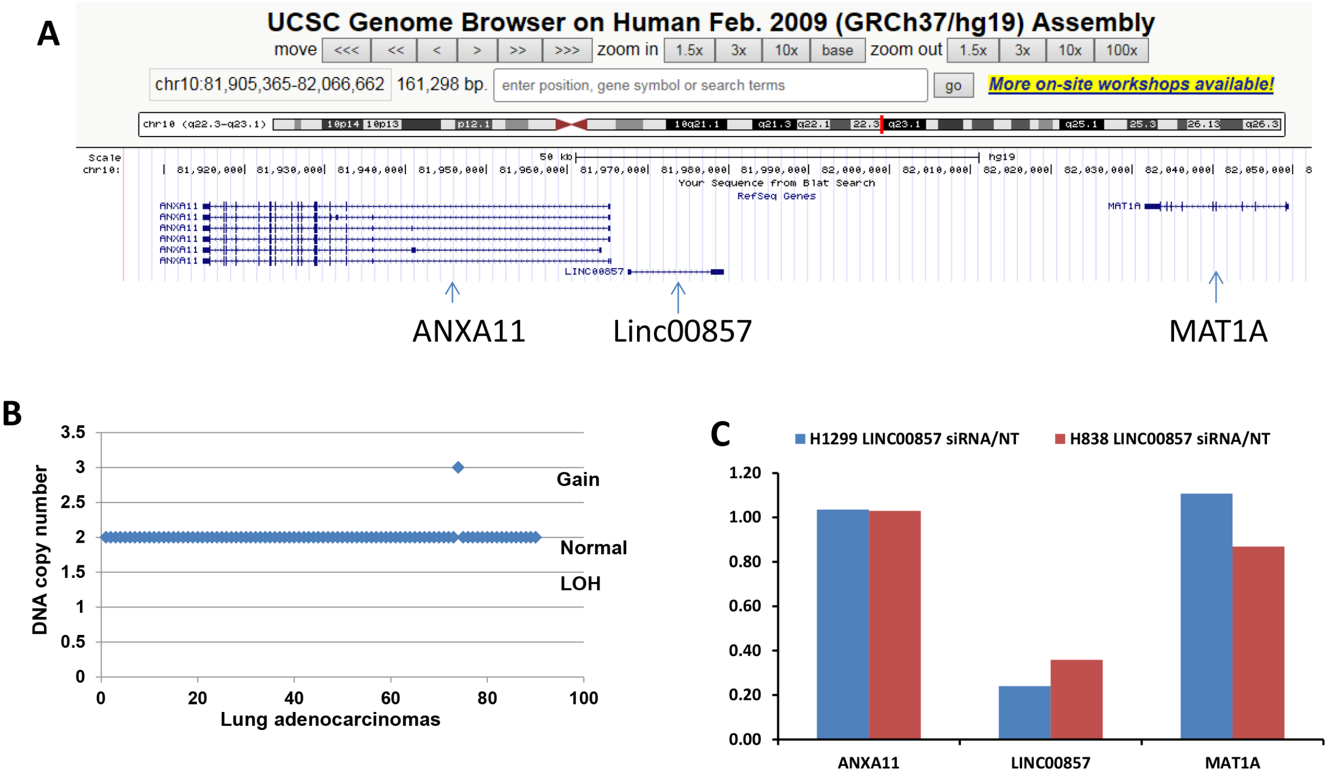

Supplementary Figure S8: (A) *LINC00857* neighbor genes *ANXA11* and *MAT1A*. (**B**) No amplification of *LINC00857* region was found as determined by Affymetrix SNP6.0 analysis on 90 lung adenocarcinomas. (**C**) Knockdown of *LINC00857* by siRNA did not affect gene expression levels of neighboring genes.

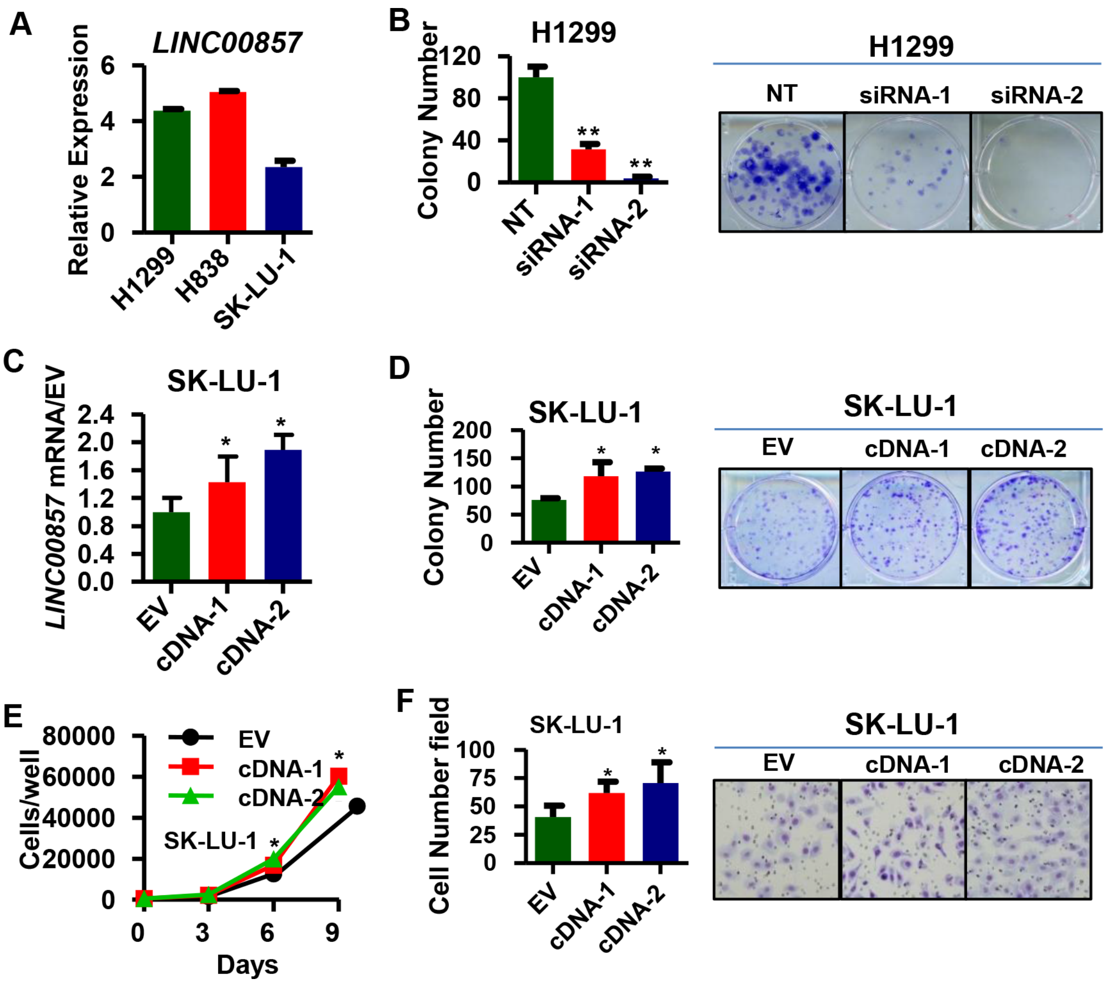

Supplementary Figure S9: (A) The relative levels of *LINC00857* in 3 LUAD cells (H838, H1299 and SK-LU-1). (**B**) Significantly decreased colony formation in *LINC00857* siRNA treated H1299 cells compared with NT Controls. $^{**}p < 0.01$. (**C**) Quantitative verification of *LINC00857* overexpression constructs in SK-LU-1 cells measured qRT-PCR.$^{*}p < 0.05$. (**D**) Increased colony formation ability after *LINC00857* overexpression compared to empty vector (EV) control. (**E**) Cell proliferation was increased upon overexpression of *LINC00857* in SK-LU-1 cells.$^{*}p < 0.05$. (**F**) Cell invasion was increased after overexpression of *LINC00857* measured by Boyden chamber matrigel assays.

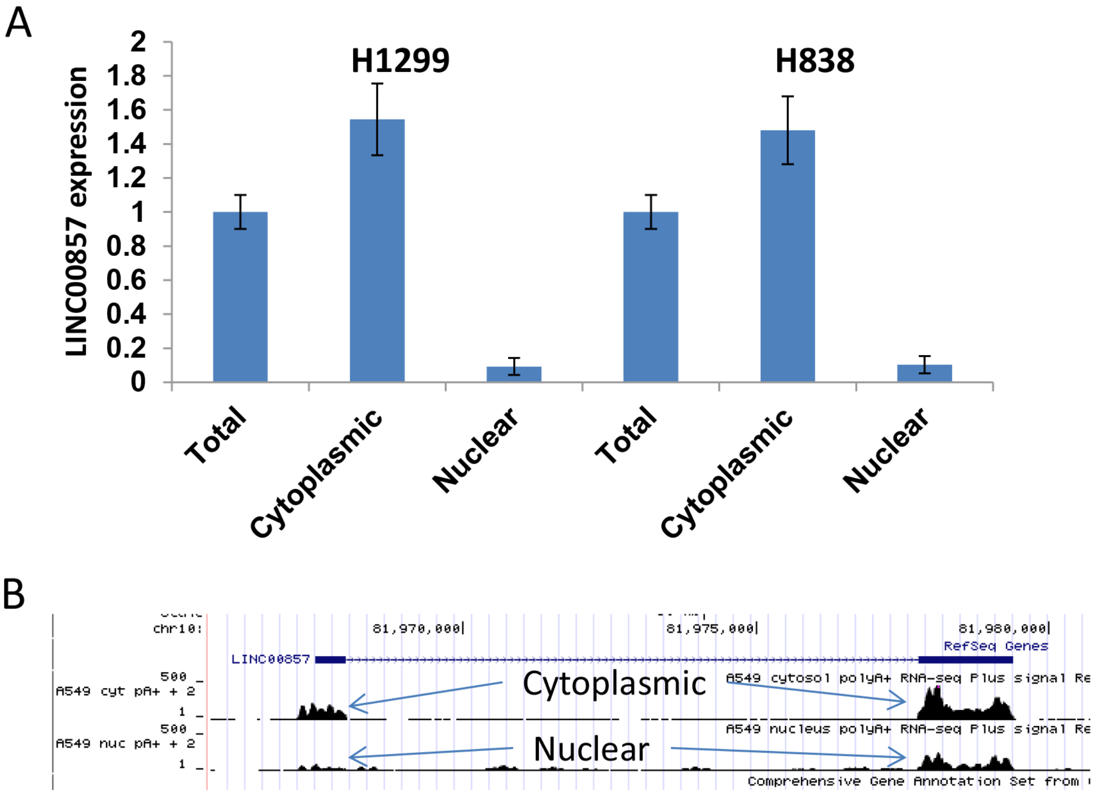

Supplementary Figure S10: (A) *LINC00857* is enriched in the cytoplasmic fraction in both H1299 and H838 cell lines measured by qRT-PCR. (**B**) Cytoplasmic enrichment of *LINC00857* in A549 measured by RNA-Seq from Encode (downloaded from UCSC browser).

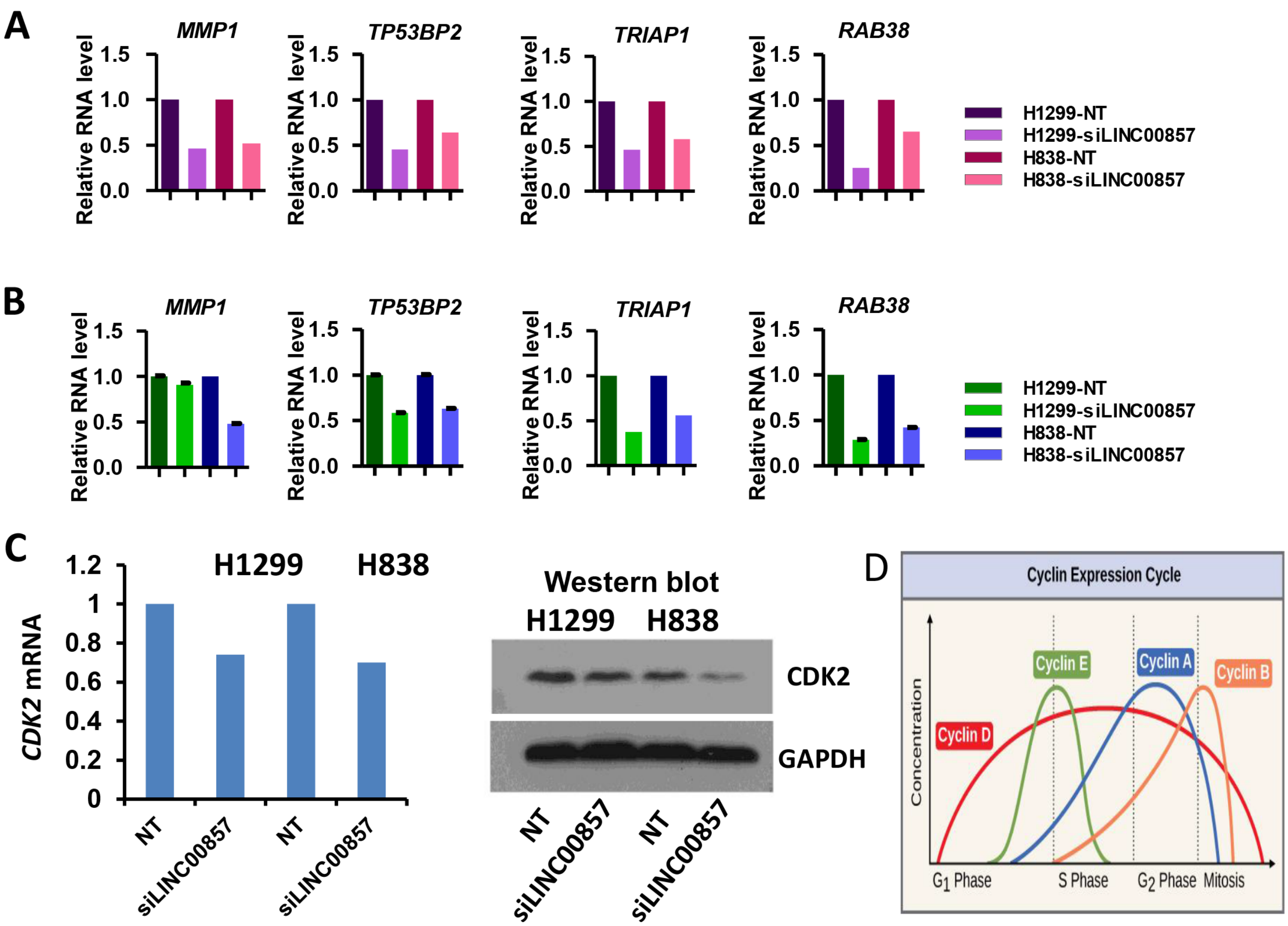

Supplementary Figure S11: (A) The expression of four genes measured by Affymetrix ST2.1 exon microarray in *LINC00857* siRNA and NT treatment on H1299 and H838 cell lines. (**B**) qRT-PCR validation of these 4 genes. (**C**) CDK2 mRNA (left) was decreased by 30% after *LINC00857* siRNA knockdown. CDK2 protein (right) was decreased on H838. (**D**) Cyclin concentrations at checkpoints. CCNE1 is highest in Gl to S phase (figure source: www.boundless.com).

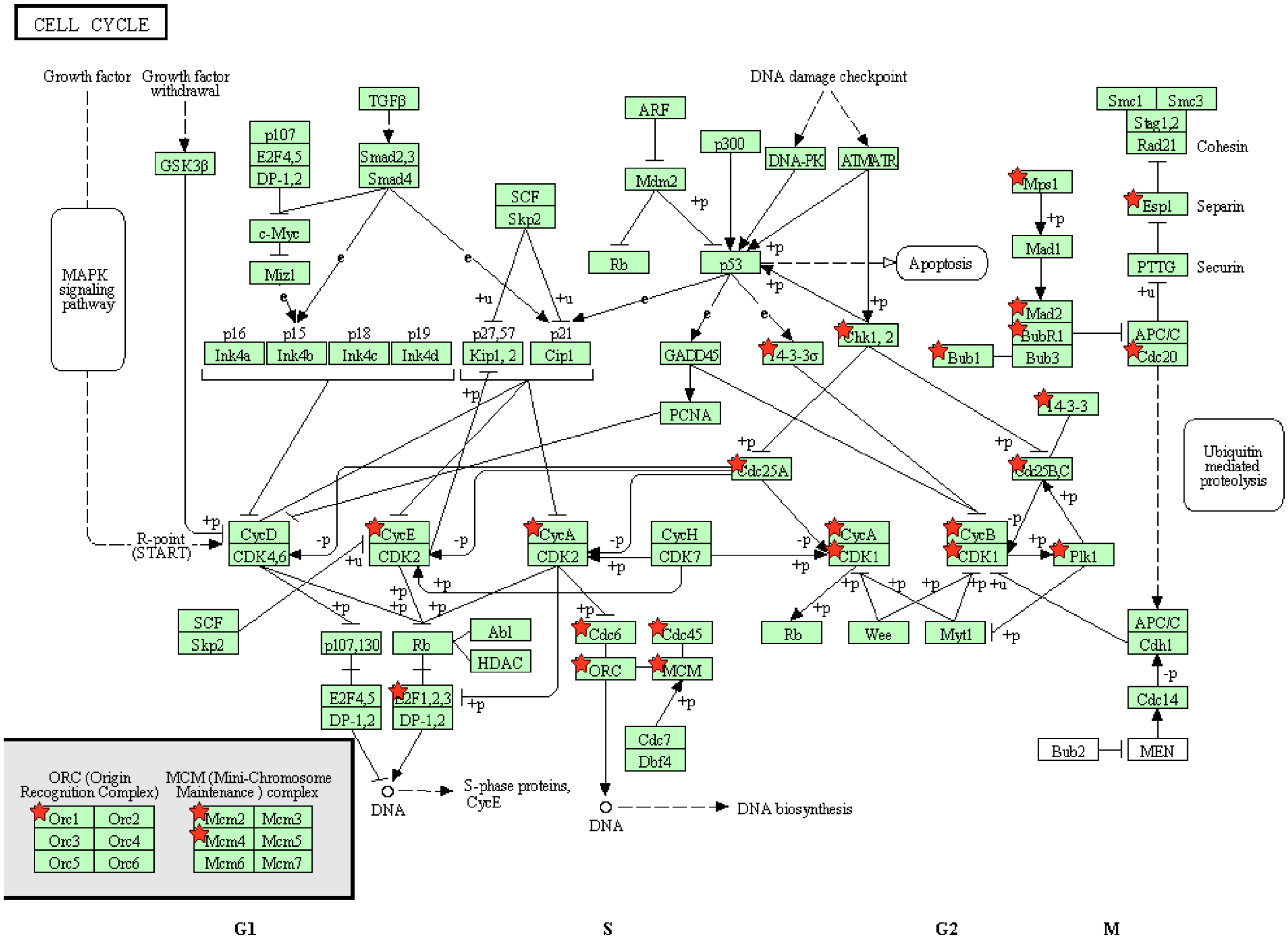

Supplementary Figure S12: Pathway analysis of *LINC00857* positively correlated genes. Red star indicates genes positively correlated to *LINC00857* expression in Okayama data set.

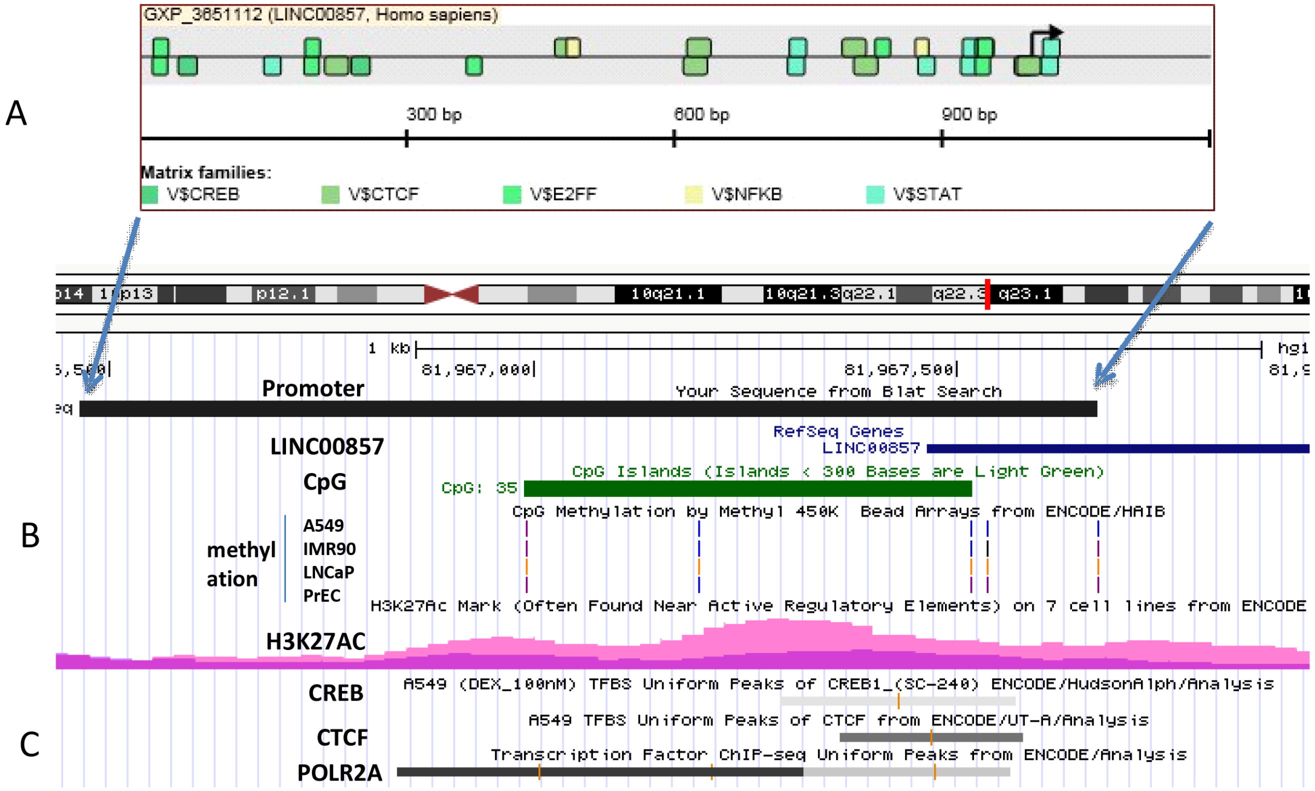

Supplementary Figure S13: ***LINC00857*** **promoter region reveals the presence of DNA binding elements for transcription factors (A and C), DNA methylation and histone acetylation (B).**

Supplementary Table S1: Characteristics of 67 lung ADs measured by RNA-Seq

Variable	Number
Age average (yrs.)	67.1
Gender	
Female	36
Male	31
Stage	
Stage I	32
Stage II	21
Stage III	14
Differentiation	
Well	14
Moderate	28
Poor	23
NA	2
Survival status	
Dead (5 yrs.)	38
Alive	29
Median follow-up (m)*	92
Recurrence	
Yes	31
No	36
Adjuvant therapy	
Yes	12
No	54
unknown	1
KRAS mutation	
Yes	31
No	36
EGFR mutation	
Yes	25
No	42
Number of mutations**	
< 5	27
> = 5	40
average	4.8

*among the patients that are still alive.
**including 23 most common oncogenes and tumor suppressor genes.

Supplementary Table S2: 281 lncRNAs with ROC > 0.7 or < 0.25 from 3 RNA-Seq data sets

		data set	UM	Seo	TCGA	UM 67LUAD 5y	UM 67LUAD 5y
		Sample ID	ROC	ROC	ROC	univariate cox	univariate cox
Ensembl ID	gene name	type	AUC	AUC	AUC	*p* value	beta
ENSG00000237523	LINC00857	processed_transcript	0.73	0.76	0.92	0.01	0.39
ENSG00000223749	AC004383.4	processed_transcript	0.80	0.88	0.73	0.04	–0.34
ENSG00000258821	AC005041.17	antisense	0.91	0.87	0.84	0.40	–0.13
ENSG00000204380	AC005042.4	antisense	0.79	0.85	0.75	0.66	–0.07
ENSG00000267073	AC005256.1	lincRNA	0.81	0.71	0.76	0.43	0.13
ENSG00000229647	AC007879.7	lincRNA	0.85	0.84	0.85	0.68	–0.07
ENSG00000267751	AC009005.2	antisense	0.82	0.85	0.83	0.05	0.37
ENSG00000259972	AC009120.6	antisense	0.73	0.86	0.84	0.87	–0.03
ENSG00000238045	AC009133.14	antisense	0.77	0.86	0.84	0.20	0.24
ENSG00000265840	AC010761.10	antisense	0.72	0.80	0.85	0.74	0.05
ENSG00000265474	AC010761.9	antisense	0.93	0.90	0.78	0.92	–0.02
ENSG00000226853	AC010894.3	lincRNA	0.70	0.72	0.75	0.84	0.03
ENSG00000236008	AC011747.4	lincRNA	0.84	0.86	0.72	0.88	0.02
ENSG00000204588	AC013268.5	processed_transcript	0.83	0.84	0.71	0.30	–0.17
ENSG00000236753	AC018642.1	processed_transcript	0.71	0.71	0.79	0.18	0.22
ENSG00000172965	AC068491.1	processed_transcript	0.81	0.75	0.83	0.05	0.32
ENSG00000258725	AC068831.8	antisense	0.93	0.92	0.87	0.75	0.05
ENSG00000234072	AC074117.10	antisense	0.97	0.91	0.81	0.64	–0.08
ENSG00000231560	AC091814.3	lincRNA	0.87	0.81	0.80	0.95	0.01
ENSG00000235493	AC092415.1	lincRNA	0.86	0.89	0.82	0.83	0.03
ENSG00000232164	AC092669.3	lincRNA	0.87	0.88	0.83	0.22	0.17
ENSG00000231890	AC093391.2	antisense	0.84	0.87	0.86	0.51	0.10
ENSG00000241181	AC093734.13	processed_transcript	0.89	0.93	0.89	0.18	0.23
ENSG00000230838	AC093850.2	lincRNA	0.92	0.87	0.89	0.01	0.39
ENSG00000265055	AC145343.2	lincRNA	0.84	0.86	0.91	0.44	–0.13
ENSG00000211454	AKR7L	polymorphic_ pseudogene	0.86	0.81	0.73	0.41	0.13
ENSG00000244676	AL109761.5	processed_transcript	0.77	0.79	0.71	0.80	0.04
ENSG00000225210	AL589743.1	lincRNA	0.84	0.79	0.84	0.23	0.20
ENSG00000234380	AP000330.8	antisense	0.83	0.91	0.89	0.97	–0.01
ENSG00000206195	AP000525.9	processed_transcript	0.84	0.79	0.85	0.15	0.24
ENSG00000236663	AP001631.9	antisense	0.71	0.81	0.86	0.85	0.03
ENSG00000236830	CBR3-AS1	processed_transcript	0.79	0.79	0.78	0.53	–0.10
ENSG00000227617	CERS6-AS1	antisense	0.91	0.82	0.80	0.22	–0.23

ENSG00000223392	CLDN10-AS1	processed_transcript	0.78	0.84	0.85	0.29	0.15
ENSG00000260655	CTA-250D10.23	lincRNA	0.75	0.85	0.73	0.09	–0.29
ENSG00000205559	CTA-384D8.20	antisense	0.74	0.79	0.73	0.13	0.25
ENSG00000266469	CTB-131K11.1	antisense	0.78	0.79	0.74	0.25	0.20
ENSG00000267696	CTB-151G24.1	lincRNA	0.82	0.83	0.77	0.49	–0.11
ENSG00000253686	CTB-43E15.3	lincRNA	0.89	0.80	0.73	0.10	0.26
ENSG00000267512	CTC-250I14.3	antisense	0.76	0.86	0.76	0.20	0.23
ENSG00000253424	CTC-436K13.3	lincRNA	0.80	0.91	0.73	0.96	–0.01
ENSG00000267575	CTC-459F4.3	processed_transcript	0.80	0.81	0.73	0.05	0.36
ENSG00000249042	CTD-2015H6.3	antisense	0.82	0.82	0.84	0.31	0.16
ENSG00000261602	CTD-2033A16.1	antisense	0.85	0.83	0.84	0.02	0.39
ENSG00000259802	CTD-2256P15.2	antisense	0.82	0.74	0.83	0.25	0.18
ENSG00000266490	CTD-2349P21.9	lincRNA	0.88	0.76	0.74	0.19	–0.21
ENSG00000265415	CTD-2510F5.4	antisense	0.90	0.76	0.89	0.01	0.37
ENSG00000265393	CTD-2517M22.17	antisense	0.71	0.83	0.80	0.35	0.15
ENSG00000260114	CTD-2574D22.4	sense_intronic	0.97	0.89	0.78	0.83	0.04
ENSG00000150316	CWC15	processed_transcript	0.77	0.74	0.77	0.01	–0.46
ENSG00000205702	CYP2D7P1	polymorphic_pseudogene	0.73	0.73	0.71	0.04	–0.33
ENSG00000226950	DANCR	processed_transcript	0.83	0.83	0.75	0.96	–0.01
ENSG00000237517	DGCR5	antisense	0.89	0.79	0.91	0.03	0.36
ENSG00000231607	DLEU2	processed_transcript	0.82	0.72	0.84	0.41	–0.14
ENSG00000204949	FAM83A-AS1	antisense	0.91	0.93	0.95	0.01	0.45
ENSG00000203499	FAM83H-AS1	processed_transcript	0.96	0.92	0.90	0.00	0.53
ENSG00000230316	FEZF1-AS1	antisense	0.96	0.94	0.90	0.09	–0.29
ENSG00000237424	FOXD2-AS1	antisense	0.95	0.93	0.90	0.80	–0.04
ENSG00000234741	GAS5	processed_transcript	0.74	0.76	0.74	0.19	0.20
ENSG00000231074	HCG18	antisense	0.78	0.86	0.90	0.09	0.28
ENSG00000228630	HOTAIR	antisense	0.87	0.73	0.75	0.83	0.03
ENSG00000253217	KB-1991G8.1	lincRNA	0.74	0.84	0.71	0.20	0.17
ENSG00000228288	KDM5B-AS1	antisense	0.99	0.93	0.93	0.44	0.13
ENSG00000257702	LBX2-AS1	antisense	0.84	0.86	0.83	0.41	–0.13
ENSG00000225880	LINC00115	lincRNA	0.95	0.91	0.78	0.83	–0.03
ENSG00000222041	LINC00152	lincRNA	0.81	0.74	0.84	0.34	0.14
ENSG00000153363	LINC00467	processed_transcript	0.84	0.95	0.85	0.24	0.19

ENSG00000248360	LINC00504	processed_transcript	0.84	0.85	0.76	0.43	0.11
ENSG00000227036	LINC00511, LINC00673	processed_transcript	0.98	0.96	0.90	0.59	0.09
ENSG00000223546	LINC00630	processed_transcript	0.79	0.83	0.79	0.77	0.05
ENSG00000232677	LINC00665	lincRNA	0.72	0.72	0.74	0.08	0.29
ENSG00000249628	LINC00942	lincRNA	0.84	0.81	0.85	0.00	0.42
ENSG00000244306	LINC01296	lincRNA	0.86	0.81	0.86	0.29	0.18
ENSG00000248538	LOC101929128	lincRNA	0.76	0.78	0.87	0.02	0.36
ENSG00000246582	LOC389641	processed_transcript	0.76	0.88	0.90	0.73	0.05
ENSG00000261801	LOXL1-AS1	antisense	0.75	0.75	0.80	0.47	0.12
ENSG00000265688	MAFG-AS1	antisense	0.76	0.76	0.81	0.12	0.24
ENSG00000147676	MAL2	processed_transcript	0.82	0.78	0.80	0.69	–0.07
ENSG00000267278	MAP3K14-AS1	antisense	0.78	0.84	0.84	0.01	0.40
ENSG00000215424	MCM3AP-AS1	antisense	0.82	0.86	0.71	0.19	0.21
ENSG00000247095	MIR210HG	processed_transcript	0.84	0.83	0.74	0.41	0.13
ENSG00000110347	MMP12	processed_transcript	0.95	0.93	0.90	0.33	–0.17
ENSG00000178814	OPLAH	processed_transcript	0.80	0.78	0.82	0.17	0.24
ENSG00000004142	POLDIP2	processed_transcript	0.73	0.81	0.91	0.00	0.52
ENSG00000146143	PRIM2	processed_transcript	0.97	0.87	0.88	0.59	–0.09
ENSG00000230082	PRRT3-AS1	antisense	0.78	0.78	0.82	0.16	0.19
ENSG00000249859	PVT1	processed_transcript	0.99	0.95	0.93	0.41	0.14
ENSG00000253722	RBM12B-AS2	antisense	0.93	0.88	0.79	0.96	–0.01
ENSG00000160957	RECQL4	processed_transcript	0.96	0.95	0.95	0.23	0.19
ENSG00000233728	RP11-109P14.9	antisense	0.79	0.89	0.90	0.50	–0.12
ENSG00000228801	RP11-110G21.1	antisense	0.73	0.83	0.73	0.14	0.25
ENSG00000246089	RP11-115C21.2	processed_transcript	0.71	0.71	0.82	0.86	0.03
ENSG00000232445	RP11-132A1.4	antisense	0.88	0.74	0.82	0.27	0.17
ENSG00000264558	RP11-138C9.1	antisense	0.77	0.92	0.83	0.06	0.29
ENSG00000251003	RP11-152P17.2	processed_transcript	0.91	0.84	0.85	0.18	0.22
ENSG00000258232	RP11-161H23.5	antisense	0.74	0.85	0.84	0.03	0.39
ENSG00000259772	RP11-16E12.2	processed_transcript	0.85	0.88	0.81	0.82	–0.04
ENSG00000244202	RP11-18A3.4	processed_transcript	0.75	0.91	0.73	0.82	0.04
ENSG00000261716	RP11-196G18.22	sense_overlapping	0.98	0.94	0.84	0.04	0.33
ENSG00000254560	RP11-1L12.3	antisense	0.90	0.81	0.90	0.84	0.03
ENSG00000261140	RP11-20I23.6	antisense	0.77	0.71	0.70	0.01	0.49
ENSG00000260877	RP11-211G23.2	lincRNA	0.74	0.72	0.75	0.80	–0.04
ENSG00000255100	RP11-21L23.3	antisense	0.78	0.76	0.72	0.49	0.11
ENSG00000231864	RP11-229P13.23	antisense	0.92	0.94	0.95	0.19	0.21

ENSG00000260442	RP11-22P6.3	antisense	0.93	0.77	0.78	0.68	–0.07
ENSG00000249731	RP11-259O2.3	lincRNA	0.90	0.89	0.83	0.66	0.07
ENSG00000259747	RP11-275I4.2	processed_transcript	0.74	0.71	0.74	0.97	–0.01
ENSG00000229953	RP11-284F21.7	antisense	0.74	0.74	0.85	0.05	0.31
ENSG00000203999	RP11-290F20.1	processed_transcript	0.79	0.88	0.92	0.29	0.16
ENSG00000249096	RP11-290F5.1	lincRNA	0.81	0.83	0.76	0.15	–0.24
ENSG00000233461	RP11-295G20.2	antisense	0.90	0.88	0.94	0.00	0.51
ENSG00000224985	RP11-297K8.2	antisense	0.78	0.88	0.85	0.25	0.18
ENSG00000261061	RP11-303E16.2	sense_intronic	0.89	0.74	0.80	0.91	0.02
ENSG00000261123	RP11-304L19.3	sense_intronic	0.87	0.76	0.87	0.49	0.11
ENSG00000241528	RP1-130H16.16	processed_transcript	0.71	0.81	0.74	0.13	0.27
ENSG00000262580	RP11-334C17.5	processed_transcript	0.73	0.79	0.72	0.26	–0.18
ENSG00000245385	RP11-334E6.10	antisense	0.73	0.73	0.75	0.20	0.23
ENSG00000235872	RP11-335O4.3	antisense	0.79	0.79	0.71	0.23	0.19
ENSG00000259380	RP11-346D14.1	lincRNA	0.84	0.87	0.78	0.72	0.05
ENSG00000257742	RP11-350F4.2	lincRNA	0.90	0.81	0.81	0.87	–0.03
ENSG00000262772	RP11-353N14.2	lincRNA	0.96	0.96	0.96	0.44	–0.13
ENSG00000253174	RP11-360L9.7	antisense	0.96	0.89	0.94	0.07	0.32
ENSG00000261373	RP11-368I7.2	antisense	0.95	0.90	0.89	0.09	0.28
ENSG00000245149	RP11-383J24.5	processed_transcript	0.92	0.89	0.76	0.10	–0.28
ENSG00000237978	RP11-385J1.2	processed_transcript	0.75	0.74	0.81	0.16	0.22
ENSG00000225518	RP11-396C23.2	processed_transcript	0.85	0.85	0.91	0.09	0.25
ENSG00000254258	RP11-398H6.1	lincRNA	0.96	0.87	0.84	0.15	–0.24
ENSG00000228437	RP11-400N13.2	lincRNA	0.85	0.82	0.76	0.52	0.10
ENSG00000261039	RP11-417E7.2	lincRNA	0.88	0.80	0.86	0.12	0.25
ENSG00000260265	RP11-44F21.5	lincRNA	0.81	0.76	0.75	0.39	0.14
ENSG00000233631	RP11-457M11.2	processed_transcript	0.73	0.75	0.74	0.50	–0.11
ENSG00000234678	RP11-465N4.4	antisense	0.84	0.88	0.83	0.56	0.10
ENSG00000260261	RP11-480A16.1	lincRNA	0.99	0.93	0.80	0.26	0.20
ENSG00000227619	RP11-492E3.2	antisense	0.75	0.88	0.90	0.00	0.48
ENSG00000263585	RP11-498C9.13	antisense	0.98	0.97	0.99	0.02	0.42
ENSG00000263198	RP11-517A5.4	antisense	0.78	0.79	0.74	0.86	–0.03
ENSG00000223478	RP11-545E17.3	antisense	0.75	0.75	0.71	0.66	0.07
ENSG00000235652	RP11-545I5.3	antisense	0.89	0.85	0.76	0.58	0.08
ENSG00000259049	RP11-589M4.1	antisense	0.79	0.82	0.93	0.02	0.44
ENSG00000259345	RP11-624L4.1	processed_transcript	0.75	0.77	0.78	0.46	–0.12
ENSG00000261762	RP11-650L12.2	antisense	0.94	0.86	0.94	0.16	0.20
ENSG00000254044	RP11-681L8.1	processed_transcript	0.72	0.75	0.87	0.13	0.21

ENSG00000249395	RP11-697M17.1	lincRNA	0.84	0.74	0.86	0.02	0.32
ENSG00000264019	RP11-6N17.6	antisense	0.73	0.90	0.81	0.92	0.02
ENSG00000249614	RP11-703G6.1	lincRNA	0.88	0.85	0.79	0.80	0.04
ENSG00000261425	RP11-709B3.2	lincRNA	0.90	0.86	0.85	0.11	0.24
ENSG00000255404	RP11-770G2.5	antisense	0.93	0.86	0.85	0.19	0.23
ENSG00000256940	RP11-783K16.5	antisense	0.97	0.92	0.96	0.00	0.50
ENSG00000248774	RP11-798M19.3	antisense	0.91	0.95	0.93	0.76	0.05
ENSG00000254721	RP11-805J14.5	antisense	0.85	0.78	0.89	0.91	0.02
ENSG00000257732	RP11-818F20.5	antisense	0.83	0.81	0.80	0.27	0.16
ENSG00000258757	RP11-841O20.2	antisense	0.88	0.93	0.95	0.29	0.18
ENSG00000224081	RP11-86H7.1	lincRNA	0.87	0.92	0.87	0.08	0.28
ENSG00000259439	RP11-89K21.1	antisense	0.77	0.86	0.83	0.08	0.30
ENSG00000254338	RP11-909N17.3	antisense	0.79	0.72	0.73	0.26	–0.21
ENSG00000253103	RP11-946L20.4	lincRNA	0.78	0.87	0.75	0.69	0.06
ENSG00000262468	RP11-95P2.1	lincRNA	0.92	0.89	0.81	0.62	–0.08
ENSG00000257596	RP11-968A15.2	antisense	0.83	0.87	0.77	0.78	–0.05
ENSG00000203288	RP11-98D18.9	antisense	0.86	0.91	0.92	0.47	0.12
ENSG00000260920	RP1-228H13.5	sense_overlapping	0.96	0.95	0.95	0.60	0.09
ENSG00000227066	RP3-340N1.2	lincRNA	0.73	0.87	0.90	0.13	–0.28
ENSG00000228274	RP3-508I15.9	antisense	0.89	0.82	0.77	0.44	0.13
ENSG00000261189	RP3-512B11.3	antisense	0.91	0.73	0.81	0.24	0.17
ENSG00000196756	RP4-564F22.2	processed_transcript	0.78	0.75	0.75	0.48	–0.12
ENSG00000213742	RP4-694B14.5	antisense	0.76	0.75	0.71	0.58	0.09
ENSG00000230798	RP4-792G4.2	antisense	0.91	0.84	0.92	0.72	–0.06
ENSG00000177788	RP5-1061H20.4	processed_transcript	0.76	0.87	0.94	0.06	0.31
ENSG00000237686	RP5-1120P11.1	processed_transcript	0.78	0.77	0.84	0.48	0.11
ENSG00000243479	RP5-1121A15.1	antisense	0.88	0.94	0.93	0.27	0.18
ENSG00000228265	RP5-1125A11.1	lincRNA	0.90	0.91	0.91	0.79	–0.04
ENSG00000224407	RP5-956O18.3	antisense	0.71	0.76	0.83	0.04	0.41
ENSG00000259153	RP6-65G23.3	lincRNA	0.91	0.93	0.94	0.51	0.10
ENSG00000229952	RP6-74O6.3	lincRNA	0.88	0.84	0.76	0.13	–0.30
ENSG00000206573	SETD5-AS1	antisense	0.83	0.88	0.71	0.25	–0.19
ENSG00000137700	SLC37A4	processed_transcript	0.75	0.94	0.90	0.75	–0.05
ENSG00000213599	SLX1A-SULT1A3	processed_transcript	0.94	0.72	0.73	0.96	0.01
ENSG00000255717	SNHG1	processed_transcript	0.91	0.88	0.85	0.87	0.03
ENSG00000242125	SNHG3	processed_transcript	0.75	0.91	0.78	0.70	–0.07
ENSG00000251022	THAP9-AS1	processed_transcript	0.81	0.74	0.73	0.51	–0.11

ENSG00000214049	UCA1	lincRNA	0.77	0.85	0.72	0.07	0.28
ENSG00000249348	UGDH-AS1	antisense	0.81	0.87	0.75	0.82	0.03
ENSG00000034063	UHRF1	processed_transcript	0.96	0.96	0.97	0.15	0.24
ENSG00000223561	AC003090.1	processed_transcript	0.16	0.17	0.21	0.12	–0.38
ENSG00000214870	AC004540.5	processed_transcript	0.10	0.17	0.22	0.18	0.22
ENSG00000267688	AC005262.2	antisense	0.25	0.20	0.20	0.02	0.43
ENSG00000239467	AC007405.6	lincRNA	0.16	0.24	0.18	0.91	–0.02
ENSG00000235584	AC008268.1	lincRNA	0.04	0.19	0.15	0.12	–0.37
ENSG00000232220	AC008440.5	antisense	0.04	0.09	0.10	0.10	0.30
ENSG00000249249	AC010226.4	antisense	0.03	0.10	0.16	0.13	–0.27
ENSG00000267107	AC011526.1	lincRNA	0.01	0.07	0.05	0.75	0.05
ENSG00000233038	AC011899.9	antisense	0.02	0.10	0.08	0.16	–0.24
ENSG00000238018	AC093110.3	antisense	0.07	0.08	0.07	0.71	0.06
ENSG00000233766	AC098617.1	antisense	0.00	0.07	0.04	0.77	0.05
ENSG00000235997	AC109642.1	lincRNA	0.01	0.11	0.11	0.76	–0.05
ENSG00000260833	AC124789.1	processed_transcript	0.19	0.10	0.06	0.22	0.19
ENSG00000263065	AF001548.6	antisense	0.06	0.22	0.15	0.39	0.14
ENSG00000151303	AGAP11	processed_transcript	0.01	0.03	0.03	0.10	–0.28
ENSG00000248445	CTB-118N6.3	antisense	0.18	0.22	0.16	0.71	–0.07
ENSG00000267598	CTC-250I14.6	antisense	0.10	0.14	0.22	0.37	0.16
ENSG00000267427	CTC-503J8.6	lincRNA	0.17	0.24	0.23	0.38	–0.13
ENSG00000259945	CTD-2012K14.3	antisense	0.10	0.14	0.11	0.31	–0.16
ENSG00000259603	CTD-2012M11.3	antisense	0.12	0.19	0.13	0.17	–0.28
ENSG00000260892	CTD-2026K11.1	antisense	0.11	0.21	0.25	0.07	0.29
ENSG00000248896	CTD-2135J3.3	processed_transcript	0.12	0.19	0.19	0.44	–0.14
ENSG00000259285	CTD-2330J20.2	antisense	0.12	0.15	0.20	0.10	–0.38
ENSG00000254429	CTD-2562J17.7	antisense	0.09	0.11	0.12	0.28	–0.19
ENSG00000254963	CTD-2562J17.9	antisense	0.08	0.24	0.16	0.66	–0.07
ENSG00000254109	CTD-3107M8.4	antisense	0.09	0.22	0.16	0.96	–0.01
ENSG00000258498	DIO3OS	lincRNA	0.05	0.23	0.16	0.92	0.02
ENSG00000225733	FGD5-AS1	antisense	0.20	0.15	0.21	0.31	0.18
ENSG00000237786	GFOD1-AS1	antisense	0.22	0.20	0.12	0.93	0.02
ENSG00000235385	GS1-600G8.5	lincRNA	0.06	0.11	0.05	0.34	–0.17
ENSG00000248890	HHIP-AS1	processed_transcript	0.05	0.14	0.15	0.79	–0.04
ENSG00000230530	LIMD1-AS1	processed_transcript	0.12	0.18	0.11	0.55	–0.10
ENSG00000196972	LINC00087	lincRNA	0.17	0.21	0.17	0.20	0.21
ENSG00000229645	LINC00341	lincRNA	0.01	0.07	0.05	0.02	–0.44

ENSG00000234456	MAGI2-AS3	processed_transcript	0.04	0.15	0.08	0.51	0.11
ENSG00000249669	MIR143HG	processed_transcript	0.14	0.23	0.20	0.93	–0.02
ENSG00000186594	MIR22HG	lincRNA	0.00	0.06	0.04	0.80	–0.04
ENSG00000267532	MIR497HG	antisense	0.02	0.10	0.10	0.37	–0.16
ENSG00000247809	NR2F2-AS1	antisense	0.04	0.12	0.12	0.23	0.18
ENSG00000224597	PTCHD3P1	antisense	0.09	0.13	0.17	0.09	0.24
ENSG00000243352	RN7SL8P	misc_RNA	0.07	0.20	0.10	0.06	–0.36
ENSG00000259884	RP11-1100L3.8	lincRNA	0.03	0.14	0.17	0.22	0.20
ENSG00000246430	RP11-16M8.2	lincRNA	0.00	0.05	0.06	0.97	0.01
ENSG00000257524	RP11-203J24.9	processed_transcript	0.09	0.23	0.22	0.13	–0.23
ENSG00000177406	RP11-218M22.1	antisense	0.12	0.23	0.21	0.70	–0.07
ENSG00000225032	RP11-228B15.4	antisense	0.07	0.16	0.10	0.20	–0.24
ENSG00000233895	RP1-122P22.2	lincRNA	0.24	0.22	0.22	0.49	0.10
ENSG00000253821	RP11-246K15.1	lincRNA	0.06	0.16	0.16		
ENSG00000228401	RP11-251M1.1	antisense	0.03	0.11	0.07	0.39	–0.15
ENSG00000258844	RP11-259K15.2	lincRNA	0.04	0.23	0.20	0.08	–0.37
ENSG00000256013	RP11-27M24.1	antisense	0.00	0.06	0.02	0.14	0.26
ENSG00000234531	RP11-288G11.3	lincRNA	0.22	0.15	0.13	0.60	0.08
ENSG00000224397	RP11-290F20.3	lincRNA	0.01	0.15	0.06	0.28	0.19
ENSG00000267774	RP11-2N1.2	antisense	0.01	0.04	0.10	0.13	0.29
ENSG00000258647	RP11-304N14.2	lincRNA	0.15	0.22	0.16	0.02	–0.54
ENSG00000225329	RP11-325F22.5	lincRNA	0.04	0.19	0.12	0.08	–0.34
ENSG00000235387	RP11-327L3.1	processed_transcript	0.04	0.08	0.07	0.27	–0.28
ENSG00000267280	RP11-332H18.4	antisense	0.03	0.09	0.07	0.48	–0.11
ENSG00000227010	RP11-336K24.6	processed_transcript	0.10	0.21	0.11	0.30	–0.19
ENSG00000257474	RP11-359M6.1	lincRNA	0.11	0.17	0.22	0.64	–0.08
ENSG00000239268	RP11-384F7.2	processed_transcript	0.02	0.04	0.06	0.27	–0.30
ENSG00000231187	RP11-38L15.3	antisense	0.15	0.18	0.09	0.37	–0.15
ENSG00000261685	RP11-401P9.4	lincRNA	0.03	0.09	0.07	0.66	–0.09
ENSG00000242569	RP11-435B5.3	lincRNA	0.11	0.18	0.14	0.79	–0.05
ENSG00000259007	RP11-463J10.3	antisense	0.03	0.18	0.21	0.36	–0.14
ENSG00000262370	RP11-473M20.9	lincRNA	0.03	0.24	0.09	0.01	–0.40
ENSG00000260879	RP11-483I13.5	antisense	0.17	0.20	0.21	0.53	0.10
ENSG00000204054	RP11-492E3.1	lincRNA	0.09	0.24	0.21	0.33	0.17
ENSG00000261452	RP11-509E16.1	lincRNA	0.11	0.17	0.19	0.30	–0.18
ENSG00000260461	RP11-541N10.3	sense_overlapping	0.06	0.15	0.09	0.66	–0.08
ENSG00000260804	RP11-566E18.3	lincRNA	0.08	0.11	0.11	0.13	–0.26

ENSG00000263707	RP11-597M12.1	antisense	0.08	0.09	0.08	0.07	–0.32
ENSG00000256948	RP11-598F7.3	antisense	0.04	0.12	0.04	0.01	–0.46
ENSG00000255191	RP11-626H12.1	lincRNA	0.08	0.14	0.17	0.70	0.06
ENSG00000266283	RP11-627G18.1	antisense	0.05	0.11	0.09	0.69	0.06
ENSG00000266010	RP11-627G18.3	lincRNA	0.12	0.23	0.09	0.20	–0.23
ENSG00000242396	RP11-67L3.5	antisense	0.15	0.24	0.17	0.23	0.20
ENSG00000264299	RP11-680F20.12	antisense	0.18	0.14	0.18	0.04	–0.34
ENSG00000227220	RP11-69I8.3	antisense	0.07	0.16	0.20	0.19	0.22
ENSG00000261054	RP11-6O2.4	antisense	0.03	0.13	0.10	0.42	0.14
ENSG00000251230	RP11-701P16.5	processed_transcript	0.02	0.09	0.03	0.44	–0.13
ENSG00000255390	RP11-732A19.5	antisense	0.09	0.19	0.21	0.57	0.09
ENSG00000255471	RP11-736K20.5	antisense	0.14	0.14	0.18	0.01	0.42
ENSG00000255197	RP11-750H9.5	antisense	0.03	0.16	0.12	0.68	–0.06
ENSG00000259094	RP11-77A13.1	antisense	0.07	0.16	0.16	0.58	–0.10
ENSG00000225278	RP11-782C8.5	processed_transcript	0.10	0.21	0.17	0.94	–0.01
ENSG00000267396	RP11-845C23.3	antisense	0.06	0.10	0.09	0.23	–0.21
ENSG00000256469	RP11-856F16.2	antisense	0.04	0.12	0.07	0.90	0.02
ENSG00000245025	RP11-875O11.1	processed_transcript	0.10	0.22	0.23	0.09	–0.31
ENSG00000257894	RP1-78O14.1	lincRNA	0.03	0.07	0.11	0.71	–0.06
ENSG00000235501	RP4-639F20.1	antisense	0.14	0.16	0.14	0.66	–0.08
ENSG00000258545	RP4-755D9.1	processed_transcript	0.12	0.23	0.15	0.89	–0.02
ENSG00000267272	RP5-1052I5.1	lincRNA	0.09	0.10	0.13	0.15	–0.23
ENSG00000259716	RP5-977B1.11	antisense	0.07	0.12	0.07	0.32	0.17
ENSG00000012171	SEMA3B	processed_transcript	0.09	0.07	0.11	0.37	0.15
ENSG00000225383	SFTA1P	lincRNA	0.07	0.13	0.14	0.01	–0.43
ENSG00000255399	TBX5-AS1	antisense	0.01	0.07	0.11	0.84	0.03
ENSG00000197253	TPSB2	polymorphic_pseudogene	0.04	0.14	0.15	0.27	–0.17
ENSG00000232352	U73167.7	antisense	0.11	0.21	0.14	0.23	0.19

Supplementary Table S3: *LINC00857* expression and clinical variables from validation set of 101 ADs measured by qRT-PCR

Variables	Validation set	Linc00857	*P* value*
Age			
< = 65	42	5.8	
> 65	59	4.5	0.2
Gender			
Female	53 (52.5%)	5.4	
Male	48	4.7	0.5
Stage			
Stage I	59 (58.4%)	4.6	
Stage II	16	4.6	0.2
Stage III	26	6.4	0.2
N status			
No		4.6	
N1-2		6.1	0.3
Differentiation			
Well	28	3.3	
Moderate	38	6.7	0.005
Poor	34 (33.7%)	4.6	0.1

****t*** test was used.

Supplementary Table S4: Genes regulated by *LINC00857* siRNA knock down

ProbeID	Symbol	H1299 NT	H1299 LINC00857 siRNA	H838 NT	H838 LINC00857 siRNA	H1299 LINC00857 siRNA/NT	H838 LINC00857 siRNA/NT
16706610	LINC00857	6.03	3.97	6.16	4.68	0.240	0.359
17125850		4.43	1.60	4.03	3.26	0.141	0.586
17025664	MIR1913	3.88	1.14	2.37	1.32	0.150	0.481
16813999		3.36	0.71	2.09	1.32	0.159	0.588
17089363		3.74	1.37	2.87	1.16	0.193	0.305
16915113		3.06	0.79	2.55	1.58	0.208	0.511
16847414		3.02	1.08	1.82	0.84	0.260	0.504
17024315		3.81	1.90	1.49	0.44	0.265	0.485
16979944		5.16	3.30	2.66	1.64	0.276	0.496
17118066	LOC728093	3.43	1.63	3.80	2.88	0.288	0.530
17118127	LOC728093	3.43	1.63	3.80	2.88	0.288	0.530
17118104	LOC728093	3.43	1.63	3.80	2.88	0.288	0.530
17118058	LOC728093	3.43	1.63	3.80	2.88	0.288	0.530
16771030		2.16	0.36	1.83	0.64	0.289	0.437
16934866	MIR659	2.81	1.16	2.83	1.07	0.319	0.295
16902923	MIR663B	3.17	1.64	3.03	1.91	0.348	0.458
17005611	BTN3A2	2.20	0.70	1.76	0.98	0.352	0.584
16766940		4.58	3.09	6.06	4.57	0.356	0.356
16975928	USP46	5.26	3.78	5.55	4.45	0.359	0.469
16934552		2.07	0.59	1.69	0.75	0.359	0.518
17061757		3.85	2.38	3.45	1.64	0.361	0.284
17078914		4.83	3.36	5.56	4.74	0.361	0.566
16907324	MPP4	4.73	3.29	3.86	2.91	0.368	0.518
16824123		4.82	3.39	4.76	3.78	0.373	0.508
16818473		2.32	0.90	2.15	1.20	0.373	0.517
17118399		2.24	0.83	1.81	0.81	0.374	0.499
16773224	C1QTNF9	2.05	0.65	1.66	0.74	0.378	0.530
16850090	DUS1L	6.38	5.02	5.55	4.12	0.390	0.370
16698120		4.27	2.93	4.71	3.65	0.396	0.479
16869058		4.36	3.03	3.18	0.90	0.398	0.207
16799492	LOC100505573	5.66	4.33	3.71	1.64	0.399	0.238
16824572	GPRC5B	2.26	0.95	3.92	2.69	0.403	0.425
17123882		6.52	5.22	6.26	5.50	0.405	0.592
16701911		2.00	0.72	2.55	1.13	0.411	0.372
16968378	ENOPH1	7.77	6.49	7.18	6.20	0.411	0.508
16869487		5.09	3.81	5.12	4.11	0.413	0.494
16945868		2.27	1.00	1.96	0.92	0.415	0.489

16957565		3.07	1.81	5.40	3.71	0.415	0.309
17108827	PRKX	6.92	5.65	5.50	4.11	0.416	0.381
16969700	GAR1	5.44	4.18	5.16	3.93	0.416	0.426
16944931	FAM86JP	3.09	1.83	3.34	2.57	0.417	0.588
16860418	CCNE1	6.76	5.51	6.79	5.75	0.421	0.484
17085073		2.23	0.99	3.73	1.42	0.422	0.201
17009902		3.33	2.10	1.72	0.87	0.424	0.557
16793721	PPP2R5E	7.96	6.78	7.71	6.79	0.441	0.529
17067755	MAK16	7.81	6.63	7.71	6.88	0.442	0.560
17016486	HIST1H2BL	5.27	4.10	4.00	2.75	0.444	0.423
16678907	COA6	5.22	4.06	4.84	3.95	0.448	0.540
17119158		2.40	1.25	2.55	1.25	0.449	0.407
17100862		3.61	2.46	3.32	2.39	0.451	0.528
16800678		3.03	1.89	4.13	3.01	0.452	0.461
17003208	LMAN2	9.35	8.21	8.55	7.63	0.453	0.530
16851697		1.93	0.79	1.57	0.80	0.453	0.587
16684089	RPA2	6.65	5.52	6.48	5.55	0.457	0.524
16743721	MMP1	2.45	1.33	2.00	1.05	0.462	0.520
16808386	MFAP1	8.64	7.52	8.75	7.94	0.462	0.568
16771303	TRIAP1	5.50	4.39	4.74	3.95	0.463	0.579
16855578	LMAN1	9.84	8.73	9.81	8.71	0.463	0.469
17065851	FAM66D	3.58	2.49	3.38	2.14	0.471	0.421
17079436	HRSP12	6.22	5.17	5.67	4.85	0.482	0.567
16855684	KDSR	7.92	6.87	7.04	6.02	0.483	0.494
16732043	MIR4492	3.47	2.42	5.01	3.96	0.483	0.482
16840284	C1QBP	9.74	8.70	9.58	8.68	0.485	0.537
16826435		3.82	2.78	3.07	2.27	0.486	0.576
17051497		3.28	2.26	4.35	2.40	0.492	0.259
17118022		4.65	3.65	5.19	4.25	0.500	0.520
16994942		1.87	0.87	2.11	0.70	0.501	0.377
16755221	LOC144486	3.31	2.31	3.85	2.57	0.501	0.413
16666545	LPHN2	5.59	4.60	5.88	4.54	0.503	0.394
17047382	TRIM73	3.40	2.42	2.27	1.06	0.506	0.434
16960618	TMEM14E	1.98	0.99	2.64	1.64	0.506	0.503
16989259		2.58	1.60	2.66	0.97	0.507	0.311
17092118		2.09	1.12	1.86	0.94	0.510	0.526
17005535	HIST1H4A	2.72	1.75	3.65	2.88	0.513	0.589
16836824	MRC2	3.14	2.18	3.52	2.49	0.514	0.490
16769838	MIR619	2.75	1.79	2.51	1.39	0.514	0.461

16837187		2.13	1.19	1.91	1.06	0.523	0.555
17048072	STEAP1	5.83	4.90	3.07	0.99	0.525	0.236
17089982	DOLPP1	7.20	6.27	6.69	5.78	0.527	0.534
17043365		4.75	3.83	5.03	4.24	0.527	0.578
16846283	HOXB8	2.38	1.46	2.59	1.48	0.527	0.465
16979845		3.38	2.46	3.22	1.83	0.528	0.382
16968428		5.89	4.98	4.56	3.69	0.530	0.549
16661459	STX12	6.25	5.33	6.41	5.42	0.531	0.500
16944432	MAATS1	4.59	3.68	1.91	1.10	0.532	0.569
16870733	ZNF14	4.98	4.07	2.90	1.60	0.532	0.405
17046737		4.12	3.21	2.88	1.29	0.533	0.334
16914941	RP4-724E16.2	1.82	0.92	3.68	2.20	0.536	0.357
17126216		3.36	2.47	3.94	3.16	0.538	0.583
17068061		3.00	2.11	3.76	2.92	0.540	0.561
17124364		3.82	2.94	3.55	2.60	0.543	0.519
16703659	MAP3K8	1.91	1.03	6.56	5.48	0.544	0.472
16692620		3.95	3.07	3.73	2.16	0.544	0.335
17079396		2.15	1.27	2.12	0.78	0.545	0.395
16858003		4.21	3.35	3.83	2.65	0.551	0.440
16903929	FAM133DP	4.63	3.77	4.86	3.83	0.551	0.491
16900018		6.93	6.07	7.36	6.53	0.552	0.560
16747896	CLEC4A	2.15	1.29	2.14	1.10	0.552	0.486
16695388		2.84	2.00	4.44	1.64	0.558	0.144
16937671	HRH1	2.60	1.76	4.00	2.66	0.558	0.396
17126104		3.36	2.52	3.70	2.44	0.559	0.420
16869684	EMR2	2.14	1.30	4.08	2.88	0.559	0.434
16730522	BIRC3	9.27	8.43	5.45	4.12	0.559	0.397
16666416	NEXN	2.82	1.99	6.06	5.31	0.564	0.594
16899982		1.82	1.00	2.65	1.50	0.564	0.453
17019134	USP49	3.19	2.36	2.29	1.51	0.565	0.580
17119006		3.76	2.93	4.19	2.73	0.565	0.365
16824022		2.55	1.74	3.48	1.84	0.569	0.321
16997010		1.51	0.70	2.55	1.64	0.570	0.532
16669463		1.73	0.93	2.69	0.99	0.571	0.308
17080444		2.09	1.29	3.37	2.51	0.574	0.550
16792194		1.68	0.88	1.56	0.72	0.575	0.559
16896315		2.26	1.47	2.74	0.89	0.575	0.279
16911132	PRNP	7.28	6.48	8.47	7.44	0.576	0.491
16669566		5.35	4.56	6.74	5.96	0.576	0.582

16670197		5.35	4.56	6.74	5.96	0.576	0.582
16672298	KIRREL-IT1	4.19	3.40	5.58	4.08	0.579	0.354
16934749	RAC2	1.91	1.13	2.35	1.52	0.581	0.560
17084605	UNC13B	7.11	6.33	5.91	5.08	0.581	0.561
17074735		5.04	4.26	5.33	4.57	0.582	0.589
17125782		2.26	1.49	3.27	2.39	0.585	0.544
16679970	LINC00115	1.26	0.49	1.78	0.79	0.586	0.504
17108745	DHRSX	1.37	0.60	2.66	1.42	0.587	0.422
16978502	SLC9B1	1.46	0.69	1.56	0.74	0.588	0.567
16817685	PAGR1	5.07	4.30	5.53	4.68	0.589	0.556
16761549	PRB1	2.20	1.43	3.45	1.70	0.589	0.298
16948236	ZNF639	5.33	4.57	5.72	4.73	0.590	0.503
16670291		2.62	1.86	2.78	1.76	0.592	0.496
16749103		3.95	3.19	4.56	3.59	0.592	0.513
17016025		2.18	1.43	2.56	0.71	0.593	0.277
17035334		2.53	1.78	3.24	2.21	0.593	0.490
16833295		1.66	0.91	1.92	0.82	0.594	0.469
16995534	MIR3650	2.36	1.61	3.21	2.02	0.595	0.436
16676515		3.85	3.10	3.77	2.45	0.598	0.400
17119204		1.26	0.52	2.23	1.42	0.599	0.569
17106179	ACSL4	2.62	1.88	3.02	1.92	0.600	0.466
16701630	OR2T35	0.63	1.40	0.56	1.63	1.700	2.106
16775162	DIAPH3-AS2	0.56	1.34	0.69	2.12	1.718	2.710
16704298		0.94	1.73	1.30	4.09	1.722	6.906
16889633		1.53	2.32	2.36	3.68	1.728	2.492
16885221		1.99	2.78	3.03	4.04	1.733	2.013
16926027		2.90	3.69	3.40	4.36	1.735	1.945
16901533		7.22	8.01	2.13	3.31	1.736	2.264
16971251		1.85	2.65	1.66	3.74	1.744	4.234
17065905	MIR3926-2	1.29	2.10	1.29	2.23	1.746	1.922
17079348	LOC100500773	4.73	5.53	5.07	5.94	1.747	1.833
16758128		0.44	1.25	0.86	1.80	1.753	1.911
17117480	LOC100507663	3.08	3.91	4.43	5.37	1.775	1.919
16930182	APOBEC3F	2.11	2.94	1.38	2.92	1.782	2.913
16983742		1.68	2.51	1.92	3.49	1.783	2.961
16921973	MAP3K7CL	4.29	5.13	2.92	4.54	1.790	3.059
17065630	MIR597	2.67	3.52	3.33	4.98	1.797	3.140
16764833	KRT83	0.95	1.81	1.34	2.13	1.808	1.730
16864934	ZNF525	2.47	3.32	1.90	3.26	1.813	2.566

16822265		3.44	4.30	3.02	3.83	1.825	1.747
16875862	ZSCAN5A	4.56	5.44	4.75	6.06	1.834	2.478
16816895		5.25	6.12	6.45	7.39	1.834	1.918
16824899		5.25	6.12	6.45	7.39	1.834	1.918
16824980		5.25	6.12	6.45	7.39	1.834	1.918
16818543		1.54	2.42	1.17	2.62	1.848	2.718
16770780		2.87	3.76	5.26	6.16	1.854	1.869
16913664		2.44	3.33	2.56	4.02	1.857	2.759
17118390	MGC24103	2.95	3.85	4.05	4.90	1.861	1.795
16993286		1.46	2.38	1.45	2.39	1.890	1.921
16975456		2.63	3.57	2.44	3.85	1.912	2.673
16696612		0.98	1.92	1.09	2.00	1.912	1.880
17118734		0.98	1.92	1.09	2.00	1.912	1.880
16898108	SNORA70B	3.60	4.54	3.60	5.02	1.912	2.676
17005991		0.62	1.56	1.21	2.32	1.918	2.149
16714341		2.95	3.89	3.36	5.30	1.918	3.848
16797978		1.03	1.98	1.31	2.25	1.929	1.913
16761502	TAS2R14	5.18	6.14	5.20	6.23	1.935	2.050
17036177		1.25	2.21	1.61	2.83	1.938	2.330
16960149	PLSCR4	3.03	3.99	4.00	4.85	1.943	1.800
17022916	FRK	1.96	2.92	3.74	4.79	1.946	2.073
17013195		1.77	2.73	1.38	2.16	1.948	1.712
17119472		1.77	2.73	1.38	2.16	1.948	1.712
17114470	CT45A6	0.94	1.90	1.89	3.32	1.952	2.692
17123334		2.32	3.29	1.64	3.25	1.960	3.066
16763215		4.53	5.51	0.77	3.38	1.973	6.078
17020748	KHDC1L	3.32	4.30	3.60	4.36	1.977	1.702
16861553	ZNF570	5.28	6.27	2.93	3.71	1.986	1.714
17112187	MIR421	5.00	6.01	5.87	6.65	2.002	1.711
16739229		2.71	3.71	2.40	4.35	2.012	3.842
17011939	FAM26F	0.98	1.99	1.36	3.22	2.013	3.616
17111545	MTRNR2L10	0.79	1.82	1.18	2.59	2.044	2.649
17050000		0.65	1.68	0.77	1.58	2.051	1.755
16716525	CPEB3	3.35	4.39	3.83	4.70	2.054	1.836
16797834		0.92	1.98	1.25	3.20	2.085	3.862
16686536	RPS15AP10	3.87	4.93	4.53	5.51	2.086	1.968
16732788		0.15	1.22	0.59	1.40	2.100	1.748
17118228	HLA-E	2.38	3.45	1.22	2.09	2.109	1.826
17118232	HLA-E	2.38	3.45	1.22	2.09	2.109	1.826

17118242	HLA-E	2.38	3.45	1.22	2.09	2.109	1.826
17118246	HLA-E	2.38	3.45	1.22	2.09	2.109	1.826
17078399		0.43	1.52	2.17	3.78	2.122	3.066
16705747		1.01	2.10	0.66	2.20	2.125	2.909
17125892		3.98	5.08	3.88	4.82	2.144	1.913
17125914		3.98	5.08	3.88	4.82	2.144	1.913
17007603	MIR3934	0.83	1.94	1.18	2.29	2.158	2.161
17064088		0.76	1.88	0.54	1.60	2.166	2.074
17119680		0.76	1.88	0.54	1.60	2.166	2.074
16992622	MSX2	2.86	3.98	2.26	3.17	2.170	1.887
17065040		3.46	4.58	2.12	3.68	2.171	2.949
17059352		0.93	2.06	1.64	2.50	2.185	1.820
16917543		1.00	2.12	0.38	1.15	2.185	1.711
16835045	LRRC37A	3.74	4.87	3.96	4.83	2.201	1.837
17123102		1.54	2.68	4.46	5.55	2.202	2.137
16688162		0.62	1.77	0.71	2.48	2.224	3.422
16662749		2.30	3.46	1.67	2.93	2.226	2.388
16908478		1.10	2.26	1.30	2.24	2.233	1.909
16727322		4.10	5.29	6.98	7.82	2.282	1.798
16872528	CYP2A7	1.63	2.83	1.17	2.07	2.303	1.865
16793674		4.55	5.78	1.12	1.94	2.341	1.772
16902809	LOC100216479	0.84	2.07	0.96	2.28	2.342	2.486
16733591		2.15	3.41	1.79	2.91	2.396	2.177
17064600		3.22	4.48	4.69	5.73	2.401	2.057
17118222		1.24	2.53	1.09	2.45	2.447	2.572
16691877		1.44	2.73	5.47	6.24	2.451	1.708
16692446		1.44	2.73	5.47	6.24	2.451	1.708
16668815		1.37	2.67	3.56	5.20	2.468	3.111
16994424		5.59	6.89	1.78	3.48	2.474	3.256
17126014		1.07	2.40	0.98	2.33	2.513	2.543
16826102		3.11	4.44	2.68	4.03	2.516	2.563
16870746	ZNF506	3.82	5.16	1.60	2.99	2.533	2.635
17095177		0.74	2.16	0.92	2.14	2.677	2.336
16842143	FAM106A	0.82	2.25	0.76	1.84	2.696	2.113
17020520	LGSN	1.14	2.62	1.78	4.02	2.796	4.727
16994209		1.10	2.60	0.73	1.77	2.829	2.059
16805942	GOLGA8DP	0.93	2.45	0.75	1.75	2.860	1.996
16720728	LOC100505570	2.34	3.87	2.17	3.88	2.891	3.275
17119960		2.57	4.12	3.70	5.26	2.930	2.946

17125102		0.97	2.57	1.16	2.03	3.019	1.820
16879476		0.72	2.39	1.58	2.94	3.175	2.576
17011812		2.13	3.84	1.88	4.28	3.273	5.262
16747150		2.52	4.25	3.07	4.25	3.326	2.266
16888801		1.00	2.81	1.57	4.00	3.489	5.379
17070379		0.56	2.36	1.05	2.02	3.494	1.948
16853472		4.05	5.88	1.08	2.00	3.556	1.891
17043586		1.07	2.92	1.74	3.94	3.609	4.604
17029100		2.21	4.15	1.88	2.84	3.837	1.947
17041881		2.21	4.15	1.88	2.84	3.837	1.947
16917434		0.68	2.64	2.33	3.41	3.896	2.115
16827592		1.91	3.92	2.80	3.63	4.031	1.786
17079906		3.36	5.42	6.08	6.86	4.166	1.718
17005884	OR2B6	2.03	4.09	1.92	2.78	4.172	1.813
17117888	ZEB2	1.98	4.05	3.16	4.25	4.201	2.136
16904484	SNORA70F	0.82	3.00	1.37	2.72	4.515	2.544
16895952		1.99	4.19	1.77	3.39	4.586	3.073
17106679		1.52	3.79	2.36	3.55	4.829	2.286
17119386		1.03	3.66	0.72	1.73	6.200	2.020
16968521	MIR4451	0.99	4.37	0.72	1.63	10.397	1.887

Supplementary Table S5: Cell cycle related genes down-regulated after *LINC00857* siRNA knock down in both H1299 and H838 cells

CCNE1
CDK2
CTCFL
G0S2
MAP3K8
MAPK12
PPP1CC
PPP3CA
RBBP8
SETD8
SKA1
SPIN1
TP53BP2
UHMK1

Supplementary Table S6 : Pearson correlation of *Linc00857* and *CCNE1* and *CDK2* in primary lung adenocarcinomas

	n	CCNE1	*p*	CDK2	*p*
Okayama data	226	0.52	< 0.0001	0.46	< 0.0001
UM RNA-Seq data	67	0.48	< 0.0001	0.29	0.02

Supplementary Table S7: qRT-PCR primers

Gene name		Sequence			
LINC00857	Forward	CCCCTGCTTCATTGTTTCCC			
	Revise	AGCTTGTCCTTCTTGGGTACT			

Supplementary Table S8: siRNA

Gene name		Sequence			
LINC00857-1	sense	GGUAAGGGAAGGUGGAGAAUU			
	antisense	UUCUCCACCUUCCCUUACCUU			
LINC00857-2	sense	GGCUAUGUGCUGUGAACAAUU			
	antisense	UUGUUCACAGCACAUAGCCUU			
